# Zinc increases stress granule formation by promoting RNA condensation

**DOI:** 10.1016/j.celrep.2026.117870

**Published:** 2026-08-24

**Authors:** Luisa Macedo de Vasconcelos, Nina Ripin, Roy Parker

**Affiliations:** 1 Department of Biochemistry, University of Colorado Boulder, Boulder, CO 80309, USA; 2 Howard Hughes Medical Institute, Chevy Chase, MD 20815, USA; 3 Regensburg Center for Biochemistry, University of Regensburg, 94053 Regensburg, Germany; 4 Lead contact

## Abstract

During cellular stress, mRNAs are condensed into stress granules through the action of G3BP1 and G3BP2. How intracellular conditions affect RNA-protein condensation in stress granules is still unclear. Herein, we present several observations that cells modulate intracellular zinc concentrations to reduce the direct impact of zinc on RNA condensation. We show that oxidative stress increases the intracellular labile zinc and the expression of zinc-sequestering proteins, metallothioneins. Increased intracellular zinc leads to increased stress granule formation and delays stress granule disassembly without increasing translational repression, while zinc depletion decreases stress granule formation, demonstrating that even endogenous levels of free zinc can affect granules. Mechanistically, we demonstrate how zinc promotes stress granule formation by directly stimulating RNA condensation interactions at 100× lower concentrations than magnesium. Together, these data indicate that zinc modulates RNA condensation and stress granule formation and implies an unappreciated potential role for zinc in modulating intracellular RNA structures and interactions.

## In brief

How the intracellular environment affects RNA-protein assemblies is still murky. Macedo de Vasconcelos et al. show that zinc and similar trace metals can directly modify a cell’s ability to form stress granules. This implicates zinc in the direct modulation of RNA dynamics.

## Graphical Abstract

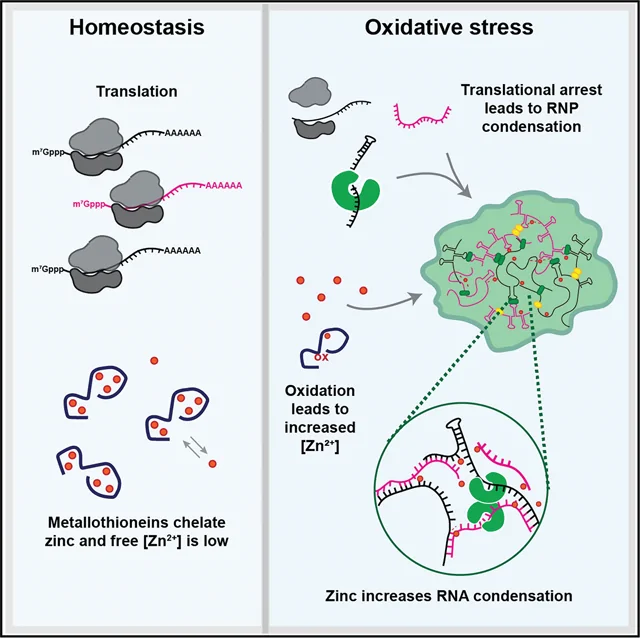

## INTRODUCTION

Ribonucleoprotein (RNP) granules are a diverse class of membrane-less condensates composed of RNAs and proteins.^1–3^ Stress granules are an RNP granule that forms during the activation of the integrated stress response when there is limited translation and an excess of free untranslating mRNAs.^1,4,5^ Stress granules are of biological interest as they affect cell survival under stress, and their formation is implicated in tumor progression and several neurodegenerative diseases.^6–9^ They are also a model system for understanding the formation and function of RNP granules.

Stress granules assemble through a combination of protein-protein, protein-RNA, and intermolecular RNA-RNA interactions. The key proteins for stress granule assembly are the paralogs G3BP1 and G3BP2, which are essential for stress granule formation under most stress conditions.^10,11^ The G3BP proteins are thought to promote stress granule assembly by forming dimers that bind to two different RNAs, thereby forming higher order RNA-protein complexes.^10–12^ Once a G3BP1-RNA co-assembly is formed, the high local concentration of RNA can trigger the formation of new intermolecular RNA-RNA interactions stabilizing the stress granule.^13^

Additional observations argue for the contribution of intermolecular RNA-RNA interactions to stress granule formation. First, RNA can self-assemble *in vitro,*^14–16^ referred to as RNA condensation, and, under approximate physiological conditions, self-assembly of total yeast RNA largely recapitulates the transcriptome of yeast stress granules.^15^ Second, DEAD-box RNA helicases have been shown to limit stress granule formation. For example, eIF4A interrupts RNA condensation *in vitro* and limits the formation of stress granules and partitioning of RNA in an ATP-dependent manner.^16^ Third, increasing free RNA by injection or transfection^17,18^ triggers stress granule formation similar to inhibiting translation initiation.^19–21^ Finally, increasing intracellular salt, which promotes RNA condensation, promotes stress granule formation, while decreasing intracellular salt, which limits RNA condensation, reduces stress granule formation.^22^

RNA condensation is affected by multiple factors. *In vitro* experiments demonstrate that positively charged small molecules such as monovalent salts, magnesium, and polyamines directly aid RNA condensation,^15,23^ presumably by shielding the negative charges on RNA. In principle, divalent cations could also promote RNA condensation by interactions with ligands on two different RNAs and thereby stabilizing the intermolecular RNA-RNA interaction. However, little work has been done examining how divalent cations affect RNA condensation in cells.

Zinc is the second most abundant trace metal in the human body and is involved in the cellular, immune, and metabolic pathways.^24–26^ It is an important enzyme co-factor, can act as a secondary molecule in immune pathways,^25^ and is involved in neuronal synapses.^27^ Zinc accumulation, specifically, has been implicated in Alzheimer’s disease pathology in multiple ways^28,29^ and type 1 diabetes’ β-cell failure.^30,31^ Zinc accumulation in β-cells triggers the integrated stress response during high-glucose treatment.^31^ These observations point toward zinc as a possible divalent cation that may be involved in stress granule formation.

In this work, we make the unexpected discovery that Zn^2+^ cations promote stress granule formation. Strikingly, Zn^2+^ ions are over 100 times more efficient that Mg^2+^ ions at triggering RNA condensation *in vitro*, which is consistent with Zn^2+^ ions’ binding to the nucleotide bases and the RNA backbone stronger than Mg^2+^ ions.^32^ This suggests that zinc promotes stress granule formation by enhancing RNA condensation, presumably by stabilizing trans interactions between RNA by liganding to two or more RNA molecules. This also raises the possibility that intracellular zinc plays unexpected roles in modulating RNA structure.

## RESULTS

### Intracellular zinc is increased during oxidative stress

Prior work has shown that oxidative stress can lead to an increase in free zinc, presumably due to zinc-binding proteins releasing bound zinc following oxidative damage.^33,34^ To study how zinc affects RNP granules, we first verified that exposure to arsenite, which generates oxidative stress, would increase free zinc in our system. Using a genetically encoded fluorescence resonance energy transfer (FRET)-based zinc reporter, ZapCV2, that reports the relative levels of free zinc^35^ (Figure 1A), we first demonstrated that the addition of 50 μM of ZnCl_2_ to the media led to a steady increase in the FRET signal, while the subsequent addition of a zinc chelator decreased the signal. Then, the ionophore zinc pyrithione was used to saturate the sensor and identify the maximum FRET signal (Figure S1A). We observed that the treatment of cells with 100–500 μM sodium arsenite led to an increase in intracellular zinc concentration (Figures 1B and 1C), which did not decline during the stress. Oxidative stress induced by 2 mM of hydrogen peroxide (H_2_O_2_) led to a transient increase in labile zinc concentrations. This may be due to H_2_O_2_ being a weaker oxidative stressor than arsenite and, as such, it was easier for the cells to return to homeostasis. In contrast, the translation inhibitor pateamine A, which inhibits translation independently of oxidative stress or the integrated stress response,^19,20^ does not lead to an increase in zinc (Figure 1B). Similarly, thapsigargin (Tg), which causes ER stress by inhibiting the calcium ATPase SERCA, does not change labile zinc concentrations. (Figure 1B). This demonstrates that oxidative stress, specifically, and not translation repression, leads to an increase in free intracellular Zn^2+^ ions.

### Metallothionein expression is increased during arsenite stress

The increase in zinc suggested that the expressions of metallothionein mRNAs and proteins might be increased following arsenite exposure. Indeed, prior proteomic analyses have shown that metallothionein proteins increase following arsenite exposure.^36^ To determine if metallothionein mRNAs increase during arsenite treatment, we examined the levels of metallothionein mRNAs by quantitative reverse-transcription PCR (RT-qPCR).

We observed that the levels of *MT1X* mRNA increased in U-2 OS cells following 1 h of 500 μM arsenite treatment (Figure 1D) but did not change in response to sorbitol stress, which also generates stress granules. In A549 cells, we also found that, at 250 μM of sodium arsenite stress, the *MT1X* mRNA significantly increased at 3 h (Figure S1C). Taken together, these results demonstrate that oxidative stress increases free zinc in mammalian cells and leads to an increase in metallothionein mRNAs, which plays a role in restoring zinc homeostasis in cells by titrating excess zinc.

### Zinc alters stress granule formation

Prior work has shown that Zn^2+^ ions strongly interact with nucleobases and phosphodiester bonds in RNAs.^37–39^ This led us to consider the possibility that zinc might interact with RNAs during stress response and affect stress granule formation. To test this hypothesis, we provided excess exogenous zinc to U-2 OS cells with or without low concentrations of sodium arsenite. Low concentrations of arsenite, such as those of 70–100 μM used here, trigger a weak oxidative stress response and generate small numbers of stress granules that are easily visible with microscopy, allowing increased sensitivity to changes in stress granule number or area.

We observed that excess zinc increased stress granule formation. In U-2 OS cells treated with 100 μM sodium arsenite, 100 μM exogenous ZnCl_2_ increased stress granule formation, while an equal concentration of exogenous MgCl_2_ did not (Figures 2A and 2B). A lower concentration of sodium arsenite, 70 μM, caused a much weaker stress response, and the effect of exogenous zinc alone was very small. However, when zinc was provided along with the ionophore pyrithione, which increases zinc cellular uptake,^40^ we observed both an increase in the number and area of stress granules and a larger percentage of cells in the population forming granules (Figures 2C, 2D, and S2A). Importantly, the addition of ionophore alone to the stressed cells did not cause an increase in stress granule formation (Figure 2C). Additionally, neither zinc nor zinc ionophore alone caused the formation of stress granules in unstressed cells (Figure S2B). Polyadenylated RNA is more enriched in stress granules in cells that are given zinc, although the ratio of RNA to G3BP1 is unchanged (Figures 2A and S2C). This argues that stress granules are still formed through the condensation of individual RNP complexes. These observations demonstrate that exogenous zinc can increase stress granule formation. In contrast, P-bodies (as determined by EDC3 foci), which are less dependent on RNA interactions for assembly,^41^ were unaffected by exogenous zinc both in unstressed and stressed conditions (Figure S2D).

In principle, an increase in stress granules can arise by increased translation repression or by increased condensation of untranslating mRNPs. To examine how increased zinc affects translation, we examined eIF2α phosphorylation and puromycin labeling to measure global translation. We did not observe any increase in eIF2α phosphorylation when exogenous zinc was provided (with or without an ionophore) (Figure S2E), nor did we observe a significant change in puromycin labeling in stress conditions (Figure 2E) or in unstressed cells treated with zinc ionophore or ionophore alone (Figure S2F). To ensure any changes in puromycin intensity are not masked by the portion of the population that does not form stress granules, we analyzed only cells with at least one stress granule. This more stringent analysis also showed no significant change in puromycin staining (Figure S2G). These data argue that zinc does not increase stress granule formation by increasing translation repression and is consistent with zinc directly affecting stress granule assembly.

We observed that stress granules disassembled slower in cells exposed to exogenous zinc. The cells without additional zinc recovered quickly such that after 1 h, an average of 38% of cells had fully cleared stress granules. (Figures 2F and S3A). We also quantified stress granule disassembly as the fraction of stress granule area. Here, the cells without exogenous zinc dissolved approximately 50% of the stress granule area in the first 30 min of recovery. (Figures 2F and 2G). In contrast, cells recovering in the presence of zinc lost only 35% of their total granule area after 30 min of recovery, and the cells treated with zinc ionophore had no disassembly after 30 min. The time point at which each population reached 50% disassembly by area was interpolated from the data and showed a significant delay between the untreated and zinc ionophore conditions (Figure 2H). We cannot confidently assess if the translation recovery with zinc was changed. We observed a modest delay in translation restoration in cells recovered with zinc (Figures S3B and S3C); however, the inherent limitations of puromycin staining did not allow us to make detailed conclusions.

Altogether, these results indicate that zinc acts as a modulator of stress granules in a manner not entirely explained by changes in translational repression.

### Metallothionein levels affect stress granule formation

The previous results suggest that the levels of intracellular zinc can increase stress granule formation, independent of changes in translation repression, potentially by strengthening the intermolecular interactions between granule components. A prediction of this model is that decreasing available intracellular zinc by overexpression of metallothioneins would limit stress granules. To test this possibility, we constitutively overexpressed MT1X and examined the formation of stress granules (Figures 3A and S4A).

U-2 OS cells overexpressing GFP-tagged MT1X formed less and smaller stress granules than GFP-expressing control cells (Figure 3B). This is also true when comparing GFP-MT1X cells having the highest GFP intensity with those having the lowest, a trend that was not seen with the GFP control cells (Figure S4B). Importantly, we observed that MT1X overexpression did not alter translation repression by puromycin labeling (Figure 3C). This indicated that the decrease in stress granule formation caused by MT1X overexpression was not due to changes in translation repression and was likely due to the chelation of zinc by MT1X. Consistent with zinc chelation reducing stress granules, we observed that the cells treated with sodium arsenite and 3 μM tris(2-pyridylmethyl) amine (TPA), a cell permeable, zinc-specific chelator, formed less stress granules than the solvent control (Figures S4C and S4D). This agrees with previously published data on the zinc chelator TPEN.^42^ These observations argue that chelating intracellular zinc by proteins or chemical means reduces stress granule formation.

We also observed that reducing metallothionein proteins has the opposite effect of increasing stress granules. In this experiment, we knocked down the transcription factor MTF1, as confirmed by western blotting (Figure 3D) and RT-qPCR (Figure 3E). MTF1 drives the expression of many metallothioneins.^43^ As expected, this caused a strong reduction of MT1X (Figure 3E). MTF1-Kd cells formed significantly more stress granules than the scramble control (siScrm) (Figures 3F and 3G). This demonstrates that reducing metallothionein proteins can increase stress granules, arguing that intracellular zinc enhances stress granule formation.

Importantly, these results show that changes to the endogenous pool of labile zinc are enough to affect stress granule formation, which implies that zinc normally contributes to stress granule formation during oxidative stress.

### Zinc stimulation of stress granule formation is independent of TIA1

Previous work suggested that zinc can promote the aggregation of the stress granule component TIA1 and that TIA1 recruitment to stress granules is enhanced by zinc.^42^ These observations raised the possibility that zinc-dependent aggregation of TIA1 could explain the increase in stress granules with enhanced zinc levels. This hypothesis predicts that in the absence of TIA1, zinc would no longer increase stress granule formation. To test this hypothesis, we successfully knocked down TIA1 to at least 33% after 72 h of small interfering RNA (siRNA) treatment, as assessed by western blotting (Figure 4A) and immunofluorescence (Figure 4B). We then examined stress granule formation in the presence or absence of exogenous zinc.

We observed that cells with decreased TIA1 showed an identical increase in stress granule formation upon the addition of exogenous zinc ionophore (Figures 4C and 4D). This observation argues that the effect zinc has on stress granules is not attributable to TIA1 protein alone. In addition, we observed that the knockdown of TIA1 had no significant impact on stress granule formation in the absence of zinc (Figure S4E), consistent with prior results that G3BP1/2 are the major proteins responsible for stress granule assembly.^10^

### Zinc directly stimulates RNA condensation

Two observations led us to hypothesize that zinc could promote stress granule formation by directly increasing RNA condensation. First, RNA folding and interactions require metal cations that mitigate electrostatic repulsion of the negatively charged backbone and promote the formation of tertiary structures^44^ as well as intermolecular interactions.^13,45^ Second, although Mg^2+^ are generally thought to be the only cations that directly bind to RNA in cells, Zn^2+^ are known to bind to RNA tighter than Mg^2+^ based on *in vitro* measurements, attributed to stronger interactions with nitrogen in bases,^32^ especially the N7 of adenine. Given this, we examined how zinc affected RNA condensation using an *in vitro* system. Specifically, we used a Cy3-labeled *in-vitro*-transcribed luciferase RNA to visualize the formation of RNA condensates in the presence of biologically relevant cations (Figures S5A and S5B). We used a buffer containing 10% polyethylene glycol (PEG) to mimic molecular crowding and 150 mM NaCl to simulate the ionic conditions of intracellular environment.^15^

A key observation was that a much lower concentration of ZnCl_2_ than that of MgCl_2_ or CaCl_2_ was required to induce RNA condensation (Figures 5A and 5B). Specifically, ZnCl_2_ induced large, asymmetric RNA assemblies at concentrations ranging from 150 to 500 μM. In contrast, MgCl_2_ induced the formation of assemblies only at 12 mM, and CaCl_2_ induced only at 10 mM (Figures 5A and 5B), approximately 100 times higher concentrations.

To determine if the condensation of RNA by zinc or magnesium was reversible, we examined if these RNA condensates disassembled following chelation of zinc or magnesium. Following RNA condensation using either 15 mM MgCl_2_ or 500 μM ZnCl_2_ (Figure S5C, top row), we added 2× molar equivalent of EDTA or TPA, a zinc-specific chelator, or the equivalent volume of DMSO (Figures S5C and S5D). As expected, RNA assemblies were dissolved by chelating the relevant divalent cation. Importantly, both TPA and EDTA were able to dissolve RNA condensates formed in the presence of ZnCl_2_, while RNA condensates formed with MgCl_2_ could be dissolved only by EDTA. Thus, zinc strongly stimulates RNA condensation in a reversible manner.

#### Zinc alters RNA-G3BP1 co-condensation

The strong effect of zinc on RNA condensation *in vitro* argues that zinc might directly affect stress granule formation by promoting RNA condensation. To examine this further, we tested how zinc affected the assembly of a minimal reconstituted stress granule produced by the co-condensation of recombinant GFP-G3BP1 and Cy3-labeled luciferase RNA.

We observed changes to the nature of RNA::G3BP1 co-condensates in the presence of zinc. GFP-G3BP1 alone formed few small, spherical foci in this buffer that were not significantly changed by the addition of divalent cations, including zinc (Figures S5E and S5F). This demonstrates that any changes to the RNA::G3BP1 co-condensates cannot be solely explained by the increased condensation of GFP-G3BP1. GFP-G3BP1 and RNA form many micron-sized spherical, liquid-like condensates (Figure 5C). The addition of 3 mM MgCl_2_ to the buffer produced slightly larger condensates (iii), but 10 mM MgCl_2_ collapsed condensates into dense, mesh-like assemblies (iv). Surface tension drives phase separations into spherical shapes as this minimizes interfacial area and the associated energetic penalty.^46^ However, condensates can also assume non-spherical morphologies driven by the formation of semi-rigid networks.^14^ The change in morphology with the increase in magnesium concentration suggests that increasing strength of interactions promote more stable structures with longer relaxation times.

Strikingly, even the lowest levels of ZnCl_2_ tested (50 μM) formed co-condensates with mesh-like morphology (v). It should be noted that zinc concentrations that gave mesh-like assemblies were two orders of magnitude lower than MgCl_2_ concentrations with similar effects (Figure 5C). Higher concentrations of ZnCl_2_ led to increasingly large, mesh-like co-condensates (vi). The formation of zinc-dependent mesh-like condensates suggests that Zn^2+^ ions tend to increase the number and/or strength of intermolecular RNA-RNA interactions.

A prediction of Zn^2+^ ions increasing RNA-RNA interactions is that zinc should lead to faster hardening of G3BP1:RNA co-condensates. Hardening of G3BP1:RNA co-condensates occurs as intermolecular RNA-RNA interactions form and render the RNA assembly stable, even after G3BP1 is degraded by protease K.^13^ The RNA portion of G3BP1 co-condensates formed with zinc becomes protease resistant earlier and with lower concentrations of divalent cation relative to those formed with Mg^2+^ (Figure S5G). Proteinase K completely degraded GFP-G3BP1, as determined by microscopy (Figure S5G). The zinc-formed condensates retained RNA assemblies, while the magnesium condensates disassembled (Figures S5G and S5H). This indicates that zinc promotes stable RNA-RNA interactions quicker and at lower concentrations compared with magnesium.

### Copper increases RNA condensation and stress granule formation

The strong effect of Zn^2+^ on RNA condensation can be explained by zinc’s stronger binding to both the bases and phosphate backbone compared with Mg^2+^.^32^ This predicts that other metal ions with strong binding to bases and the phosphate backbone would also strongly promote RNA condensation and would increase stress granule formation in cells. For example, Cu^2+^ ions exhibit strong binding to nucleobases and phosphates similar to or better than Zn^2+32,39^; thus, Cu^2+^ ions would strongly promote RNA condensation *in vitro*. Indeed, we observed that CuCl_2_ promoted RNA condensation at low concentrations similar to ZnCl_2_ (Figures 6A and 6B).

If zinc’s effects on stress granules are due, in large part, to increased condensation of RNA, we predict that Cu^2+^ ions would also stimulate stress granule formation in cells. To test this, we exposed U-2 OS cells to sodium arsenite with or without 100 μM exogenous CuSO_4_ for 1 h. Cells exposed to stress and copper sulfate formed more stress granules than cells exposed to sodium arsenite alone (Figures 6C and 6D). Importantly, copper sulfate alone, with or without the ionophore pyrithione, neither caused the formation of stress granules nor caused cell death after 1 h, indicating that the Cu^2+^ addition increased the condensation of RNPs released from translation due to arsenite stress.

The correlation of ions strongly triggering RNA condensation *in vitro* and promoting stress granule formation *in vivo* argues that these ions tend to enhance stress granule formation through RNA condensation. Because copper is a very sparse metal^47^ ion in cells, it is unlikely to affect stress granules normally and does so only in this case, as we provided sufficient exogenous Cu^2+^ to contribute to RNA condensation.

## DISCUSSION

We present several observations that the level of intracellular zinc promotes the formation of stress granules. First, we observed that free zinc increases upon arsenite exposure (Figure 1). Second, excess zinc was observed to lead to an increase in stress granule formation and reduced stress granule disassembly (Figure 2). Third, we observed that the overexpression of MT1X or use of TPA, which chelates intracellular zinc but not magnesium,^48–50^ reduces stress granule formation (Figures 3 and S4). This observation argues that the normal levels of intracellular zinc can promote stress granule formation.

Several observations *in vitro* argue that zinc promotes stress granule formation by increasing RNA condensation. First, Zn^2+^ function at ~100-fold lower concentrations than Mg^2+^ to promote RNA condensation *in vitro* (Figure 5). The ability of Zn^2+^ to promote RNA condensation more efficiently than Mg^2+^ is consistent with the observations that zinc can directly bind to mononucleotides, ATP, ssRNA, and such nucleobase-containing polymers,^32,37,39,51^ with higher affinity to nucleotide bases than magnesium, principally adenine, through the N7 of guanosine and adenine. Second, we observed that Cu^2+^ ions, which also strongly condense RNA *in vitro*, promote stress granule formation in cells (Figure 6). Third, we observed that Zn^2+^ ions promote mesh-like co-condensation of G3BP1 and RNA and that those co-condensates harden faster than in the presence of Mg^2+^ ions (Figure S4), which is consistent with stronger intermolecular RNA-RNA interactions in these co-condensates (Figure 6). Finally, we demonstrated that zinc promotes stress granule formation, even in cells deficient in TIA1, thereby ruling out that zinc promotes stress granules through TIA1 aggregation (Figure 4).^42^ Taken together, these observations strongly suggest that zinc promotes stress granule formation by enhancing RNA condensation.

The observation that zinc promotes RNA condensations *in vitro* and stimulates stress granules in cells raises the possibility that zinc has more prevalent roles in RNA folding and interactions than previously considered. Magnesium is the most accepted and well-studied divalent cation thought to affect RNA folding and interactions due to its long history as a studied cation for RNA structure and its higher intracellular abundance. Specifically, magnesium is estimated to be present at ~15 mM concentrations in cells, with 0.5–3 mM Mg^2+^ ions being freely available and most magnesium being chelated by metabolites.^52^ In contrast, total intracellular zinc concentration varies between 150 and 300 μM depending on the cell or tissue type,^24^ but the vast majority of Zn^2+^ is thought to be chelated by proteins, including cysteine-containing zinc-finger proteins.^53^ Our data would imply that some Zn^2+^ are directly bound to RNA in cells (Figure 7), but this has not been experimentally demonstrated to date. It should be noted that zinc bound to RNA would be predicted to alter RNA folding in some cases compared with magnesium since zinc exhibits stronger binding to the N7s of guanosine.^32^ This suggests that it will be important in future work to determine if and how zinc affects RNA folding in cells.

### Limitations of the study

One difficulty in studying factors that change RNP condensates is disentangling the effect on one component from the entirety. For example, we cannot rule out the formal possibility that zinc promotes stress granule formation also through RNA-binding proteins found in stress granules. Zinc-finger motifs are enriched in the stress granule proteome, and many RNA-binding proteins contain these motifs and are dependent on zinc for efficient RNA binding.^54^ For example, ZCCHC3 and ZAP are zinc-finger proteins recruited to stress granules, which might be altered by changes in zinc concentration.^55,56^ It is possible that the combined effect of zinc-deficient zinc-finger proteins could change stress granule formation under low zinc conditions for example, but it is unlikely that such proteins would be zinc deficient in normal conditions and affected by excess zinc. We also cannot rule out the combined effects of zinc binding to intrinsically disordered regions of proteins in stress granules.

It is also unclear if zinc will affect stress granules under other stresses that do not lead to an increase in intracellular zinc concentrations. Most importantly, although our observations strongly suggest that Zn^2+^ cations can bind to RNA in cells, neither this nor zinc enrichment in stress granules has been directly and experimentally observed.

## RESOURCE AVAILABILITY

### Lead contact

Additional information and resource requests should be directed to and will be fulfilled by the lead contact, Roy Parker (roy.parker@colorado.edu).

### Materials availability

Any and all reagents generated in this study are available from the lead contact without restriction.

### Data and code availability

- Due to file size constraints, any and all data are available upon request.
- This paper does not report original code.
- Any additional information required to reanalyze the data reported in this paper is available from the lead contact upon request.

## STAR★METHODS

### EXPERIMENTAL MODEL AND STUDY PARTICIPANT DETAILS

#### Cell lines and growth conditions

Wild-type parental human osteosarcoma U-2 OS cells were originally graciously provided and described by Dr. Paul Anderson’s lab (Harvard).^57^ Cell lines were grown and maintained at 37°C and 5% CO_2_ in Dulbecco’s modified Eagle’s medium (DMEM) with 10% fetal bovine serum (FBS) and 1% penicillin/streptomycin mix unless otherwise stated. Cells were tested for mycoplasma contamination routinely by the cell culture core facility and tested negative for contamination throughout the duration of the study. All experiments were done in media supplemented with serum treated with Chelex-100 (Bio-Rad) for 12 h, followed by sterile filtration to remove Chelex-100 resin. Chelex-100 resin was used to chelate excess divalent metals.^58^

### METHOD DETAILS

#### Cell line generation

To generate MT1X overexpression cell lines, GFP-tagged MT1X and GFP sequences were produced and cloned into pTwist CMV backbones by Twist Bioscience. These sequences were cloned into pLenti-EF1-Blast plasmids as described previously.^59^ Simply, GFP and GFP-MT1X sequences were PCR amplified and cloned into *XbaI/XhoI* (New England Biolabs) sites of pLenti-EF1-Blast vectors using In-Fusion (TakaraBio) cloning (primers used are listed in Table S1).

GFP and GFP-MT1X U-2 OS cell lines were generated using a lentiviral system as previously described.^60^ HEK293T cells grown to 80% confluency in T-25 flasks were cotransfected with 2.7-μg pLenti-EF1-blast constructs, 870-ng of pVSV-G, 725-ng of pRSV-Rev, and 1.4-μg of pMDLg-pRRE using 20-μL of lipofectamine 2000 (Thermo Fischer Scientific). The pVSV-G, pRSV-Rev, and pMDLg/pRRE plasmids were a gift from Dr. Sabrina Spencer. Medium was collected 48 h after transfection and filter-sterilized with a 0.45 μM filter. Then U-2 OS cells, again at 80% confluency in T-25 flasks, after aspirating the media, were transduced with 1 mL lentiviral stocks and 10 μg/mL polybrene (Millipore) for 1 at which point normal medium was added to the flask. Cells were reseeded 24 h post transfection and selected with 5 μg/mL of blasticidin (Sigma-Aldrich).

U-2 OS cells expressing NES-ZapCV2 were generated using the PiggyBac transposon system. Plasmids carrying NES-ZapCV2 in a PiggyBac vector were kindly provided by Dr. Amy Palmer.^61^ 0.5 μg of NES-ZapCV2 plasmid and 2 μg of Super PiggyBac transposase plasmid were co-transfected into U-2 OS cells (70% confluency in a 6 well plate) using Lipofectamine 3000 (Thermo Fisher Scientific) according to the standard manufacturer’s protocol for 6 well plates. Cells were selected with 400 μg/mL G418 (Thermo Fisher Scientific) for 24 h, recovered for two generations and selected again with flow cytometry.

All generated cell lines were confirmed with western blots and PCR (primers used are listed in Table S1).

#### Immunofluorescence

U-2 OS cells were seeded on sterilized coverslips (Warner Instruments) in 24 well tissue culture plates for 60–70% confluency on the day of experimentation. Cells were stressed for the specified time. For puromycinylation assays, cells were incubated at 37°C with puromycin (10 μg/mL in H_2_O, Gibco) 5 min prior to fixation. Treatments were then removed, and cells were washed with 1*X* nuclease-free PBS warmed to 37°C. Cells were fixed with 500 μL of 4% paraformaldehyde (Santa Cruz Biotechnology) for 11 min at room temperature. After fixation, cells were washed three times with PBS and permeabilized in 0.1% Triton X-100 (Thermo Fisher Scientific) solution in 1*X* PBS for 5 min. Once permeabilized cells were washed once with PBS and blocked with 5% bovine serum albumin (BSA, Sigma-Aldrich) in 1*X* PBS for 1 h. For protein detection, primary antibody was diluted in 5% BSA in 1*X* PBS and coverslips were incubated overnight at 4°C. Coverslips were washed thrice with 1*X* PBS then incubated in secondary antibody for 1 h at room temperature, protected from direct light. Antibody dilutions are listed in Table S2. Then, coverslips were washed thrice in 1*X* PBS and incubated in DAPI (Thermo Fisher Scientific) for 5 min at room temperature, washed in PBS and mounted onto slides with Prolong Glass (Thermo Fischer Scientific) mounting media.

#### Sequential immunofluorescence and RNA FISH

In the cases in which immunofluorescence would be followed RNA FISH, the immunofluorescence was performed as stated above with two exceptions: first, no blocking or BSA was used and incubations were done in 1*X* PBS and second, immunofluorescence was finished after the second antibody incubation.

Coverslips were then washed thrice in 1*X* PBS and refixed with 4% paraformaldehyde for 11 min at room temperature. Cells were washed twice in 1*X* PBS and then incubated in RNA FISH Wash A Buffer (2*X* SSC +10% formamide) for 5 min at room temperature. Samples were incubated overnight at 37°C on 31.25 nM of polyA-targeting FISH probes in hybridization buffer (10% dextran sulfate v/v, 10% formamide v/v, 2XSCC) in a hybridization chamber containing wet Whatman filter paper with parafilm on top. Coverslips were then washed three times in Wash A for 30 min at 37°C, with the second wash containing 0.5 μg/mL DAPI (Thermo Fisher Scientific) followed by one wash with Wash B (2*X* SSC), and slides were immediately prepared and imaged.

#### ZapCV2 FRET sensor

Zn^2+^ sensor cells were plated in glass-bottom 35 mm dishes or glass-bottom 96 well plates treated with 0.1% W/V poly-L-lysine (Santa Cruz Biotechnology) and grown for 24 h in zinc-limited media. Before the start of any experiment cells were incubated with 0.5 μg/mL Hoechst nuclear stain in HHBSS (1.26 mM CaCl_2_, 5.4 mM KCl, 137 mM NaCl, 1.1 mM MgCl_2_, 16.8 mM D-glucose (Sigma-Aldrich), 30 mM HEPES (Thermo Fisher Scientific) pH 7.4), washed twice with HHBSS and equilibrated in HHBSS for 30 min before experiments. All treatments were prepared in HHBSS at a 2*X* concentration. All imaging was performed in an environmental chamber with CO_2_, temperature and humidity control. Zinc sensor cells were calibrated *in situ* by first measuring the resting FRET ratio in individual cells in HHBSS imaging media, measuring the minimum FRET ratio and lastly, the maximum FRET ratio. Cells were imaged for CFP-YFP FRET (power 20%, 200 ms exposure, 200 gain), CFP (power 20%, 200 ms exposure, 200 gain) YFP (power 2% 150 ms exposure 150 gain) and DAPI (power 15% 200 ms exposure 200 gain).

For the resting condition cells were imaged every 30 s for the first five minutes and then every minute for the next ten minutes. To test ZnCl_2_ uptake in cells, ZnCl_2_ (Sigma-Aldrich) solution in HHBSS was added to the dish to a final concentration of 50 μM and imaged for the same program as the resting rate. Then, to obtain the minimum FRET ratio, cells were washed once with HHBSS and then added a final concentration of 50 μM Tris(2-pyridylmethyl) amine (TPA, Sigma-Aldrich) and imaged every 30 s for 8 min. Cells were then washed twice and added a final concentration of 0.001% saponin, and 750 nM pyrithione (Sigma-Aldrich) supplemented with 50 μM ZnCl_2_ and imaged every 30 s for 3 min then 1 frame per minute for 12–17 min to obtain the maximum FRET ratio.

For stress experiments, cells were prepared the same way and imaged in HHBSS for 10–15 min to obtain the resting FRET ratio. Then, the same volume of 2*X* treatments was added to the wells and images were obtained every minute for 75 min.

#### siRNA knockdowns

24.000 U-2 OS cells were seeded on sterilized coverslips in 24 well tissue culture plates and transfected with 10 pmol (TIA1) or 15 pmol (MTF1) Silencer Select siRNAs targeting TIA1 (#s14131) or MTF1 (pooled #s9027, #s9028 and #s9029) with Lipofectamine RNAiMAX (Thermo Fisher Scientific) following manufacturer’s instructions for 48 h. Knockdown was followed by immunofluorescence or harvesting for immunoblotting.

#### Immunoblotting

For GFP-MT1X and GFP cell lines and U-2 OS WT cell line for eIF2α blotting, cells were grown in 6 well plates and lysed with Pierce RIPA Buffer (Thermo Fisher Scientific) complemented with 1*X* Phosstop-phosphatase inhibitor (Sigma-Aldrich) and 1* cOmplete Mini EDTA-free Protease Inhibitor Cocktail (Sigma-Aldrich). Cells were lysed on ice for 20 min, centrifuged at 10,000g for 10 min and the supernatant was transferred to a new tube. siRNA treated cells were grown in 24 well plates, harvested in ice-cold 1*X* PBS, pelleted and lysed at −80°C. Laemmli sample buffer was added to samples. Samples were boiled for 10 min at 95°C and then run on pre-cast 4%–12% Bis-Tris Nu-PAGE gel (Thermo Fisher Scientific) and transferred with iBlot 2 nitrocellulose transfer stacks (Cellvis). Membranes were blocked with 5% nonfat dry milk or phosphatase-free 5% bovine serum albumin in Tris-buffered saline with 0.1% Tween 20 (Sigma-Aldrich) (TBST) for 1 h. Blocked membranes were incubated with primary antibody in TBST for 1 h at room temperature or overnight at 4°C. Antibody dilutions are listed in Table S2. Membranes were washed 3*X* with TBST and then incubated with secondary antibody at room temperature for 1 h in TBST. Membranes were once again washed 3*X* in TBST, and antibody detection was achieved with chemiluminescence substrate (Bio-Rad).

#### Total RNA extraction

Total human RNA was obtained from U-2 OS cells. Cells were grown to 80–90% confluency, washed with ice-cold PBS once and collected in 500 μL Trizol reagent (Thermo Fisher Scientific). In cases where extraction and purification were not done the day of the experiment lysates in Trizol reagent were stored at −80°C until the time for RNA extraction. For *in vitro* microscopy experiments, RNA was extracted by standard phenol/chloroform extraction followed by DNase I (Roche Life Sciences) treatment and ethanol precipitation and resuspended in UltraPure water. RNA destined for RT-qPCR was extracted using the Zymo Direct-zol Kit (Zymo) according to manufacturer’s instructions. RNA quality and concentration were determined using NanoDrop spectrophotometry.

#### *In vitro* transcription

A pre-existing DNA template encoding full length *luciferase* RNA (Promega) preceded by the T7 protomer sequence was *in vitro* transcribed according to the standard MEGAscript T7 Transcription (Thermo Fisher Scientific) kit instructions with a 4 h transcription cycle and 1 μg of DNA template. If fluorescent labeling was required, a ratio of 1 μL of UTP to 1.125 μL of Cy3 labeled UTP (Enzo Life Sciences) was used to obtain approximately 50% replacement with fluorescently labeled bases. The reaction was quenched by the addition of 1 μL TURBO DNase (Thermo Fisher Scientific) and RNA products were purified using the MEGAclear Transcription Clean-up kit (Thermo Fisher Scientific).

#### Microscopy of RNA self-assemblies

All experiments were carried out in 20 mM HEPES pH7.5, 10% PEG (Sigma-Aldrich, MW-3350) and 150 mM NaCl and varying amounts of MgCl_2_ (Sigma-Aldrich), CaCl_2_ (Sigma-Aldrich) or ZnCl_2_ as described in the main text. All buffers and salts were prepared in chelex-treated deionized water. RNAs were preheated for 45 s (>80°C) and cooled on ice. A final volume of 150 ng/μL of RNA was used for *in vitro* transcribed RNA experiments. Samples were prepared in 96 well glass-bottomed plates and incubated for 1 h at 25°C before imaging on the microscope.

#### Microscopy of RNA::G3BP1 co-condensates

Plasmids for recombinant GST-EGFP-G3BP1 protein were generously given by Dr. Paul Taylor. The construct contains an N-terminal GST tag and a TEV protease cleavage sequence and was purified as described in Parker, Tauber and Parker.^13^ GFP-G3BP1:RNA co-condensates were formed by adding 2–10 μM GFP-G3BP1 and 150 ng/μL cy3-*luciferase* RNA to condensation buffer (50 mM HEPES pH 7.5, 150 mM NaCl). Mg^2+^ and Zn^2+^ concentrations varied without changing the concentration of other reagents. Samples were prepared in PCR tubes and incubated for 10 to 30 min according to the experiment being done, then prepared on a slide for imaging. All condensation reactions were performed at room temperature using freshly thawed GFP-G3BP1. For proteinase K hardening assays, co-condensates were prepared as a master mix, incubated for 10 min and then distributed into separate tubes. After 30 min of incubation samples were treated with a final concentration of 0.1 U/μL of proteinase K (Thermo Fisher Scientific). Control samples were diluted with an equal volume of enzyme buffer (50% glycerol, 1 mM CaCl_2_, 20 mM Tris-HCl pH 7.4). Samples were incubated at room temperature for 5 min before being prepared for imaging.

#### RT-qPCR

Total RNA was reverse-transcribed using SuperScript IV First-Strand Synthesis System (Thermo Fisher Scientific) with random hexamers according to manufacturer’s instructions. qPCR was run using primers (Table S1) and LightCycler 480 SYBR Green I Master mix (Roche Life Science). Samples were run in technical triplicates and at least three biological replicates on a Bio-Rad C1000 Touch CFX96 or CFX384 real-time PCR system. *Ct* values were normalized against *Gapdh* internal control and fold change calculated as 2^−ΔΔ*Ct*^.

#### Microscopy

Microscopy was performed using the inverted Nikon Ti2 Eclipse spinning disk confocal microscope outfitted with an environmental chamber with O_2_, CO_2_, temperature, and humidity control for live cell imaging. All *in vitro* RNA and RNA::G3BP1 samples were imaged with a 60*X* NA 1.27 water immersion objective and most of the fixed U-2 OS cells were imaged with either the 60× objective or a 100× NA 1.45 oil immersion objective and an ORCA-Fusion BT sCMOS camera. Live cell FRET imaging was performed with a 20*X* NA 0.75 air immersion objective and iXon Life 897 EMCCD camera.

### QUANTIFICATION AND STATISTICAL ANALYSIS

#### FRET sensor analysis

Images were processed and analyzed using Fiji image processing package and the Bio-Formats plugin. The average intensity of each channel of a small region of interest in the cytoplasm was measured. Each value was background-corrected by drawing a region of interest in a dark region of the image and subtracting the mean fluorescence intensity of the background from the mean of the cytoplasm ROI for every channel. A random selection of at minimum 18 cells per experiment was analyzed. FRET ratios for each individual cell for every timepoint was calculated with the following equation:

$$FRET\;ratio=\frac{\left(I_{\mathrm{CFPexYFPem}}\right)\mathrm{cell}\;-\;\left(I_{\mathrm{CFPexYFPem}}\right)\mathrm{background}}{\left(I_{\mathrm{CFPexCFem}}\right)\mathrm{cell}\;-\;I\left(I_{\mathrm{CFPexCFPem}}\right)\mathrm{background}}$$

Cells that died, moved out of the ROI or were otherwise unable to obtain a stable FRET ratio and YFP intensity profile were excluded from further analysis. FRET ratios of each individual cell were normalized to the average intensity of the last five frames of the resting timepoints. Sporadically, a frame for a cell would result in a negative FRET ratio due to cell movement. These individual points were excluded. Data is presented as the average normalized FRET ratio of all cells meeting analysis criteria. All experiments were performed in at least 3 biological replicates.

#### Immunofluorescence image analysis

Fiji was used for image processing and obtaining of the maximum intensity project of a series of z-stacks. Image segmentation, masking and measurements of nucleus, cytoplasm and stress granules were obtained using CellProfiler 4.2.5–4.2.8. Segmentation of cells was blindly and manually corrected only when necessary. Stress granule segmentation was filtered to remove oversized masks. R/R-studio and GraphPad Prism were used for further analysis. Stress granule area was normalized to the area of the cytoplasm. Integrated, mean and median intensity values of each channel were obtained for all masks.

#### *In vitro* RNA image analysis

Images of *in vitro* RNA assemblies were analyzed in MATLAB. Image intensity was background-corrected using negative control images. Masking was done based on median intensity of the total image in the RNA channel. Integrated intensity, number of masks and area of masks were calculated.

### STATISTICAL ANALYSIS

Statistical analysis was performed using GraphPad Prism or R/R-studio and were performed on the mean of replicates unless otherwise described. Pairwise comparisons were performed using two-tailed Welch’s *t*-test and multiple comparisons were conducted through one-way/two-way analysis of variance (ANOVA) corrected with Tukey’s multiple comparison test unless otherwise stated.

## Supplementary Material

1

2

3

SUPPLEMENTAL INFORMATION

Supplemental information can be found online at https://doi.org/10.1016/j.celrep.2026.117870.

## Figures and Tables

**Figure 1.**
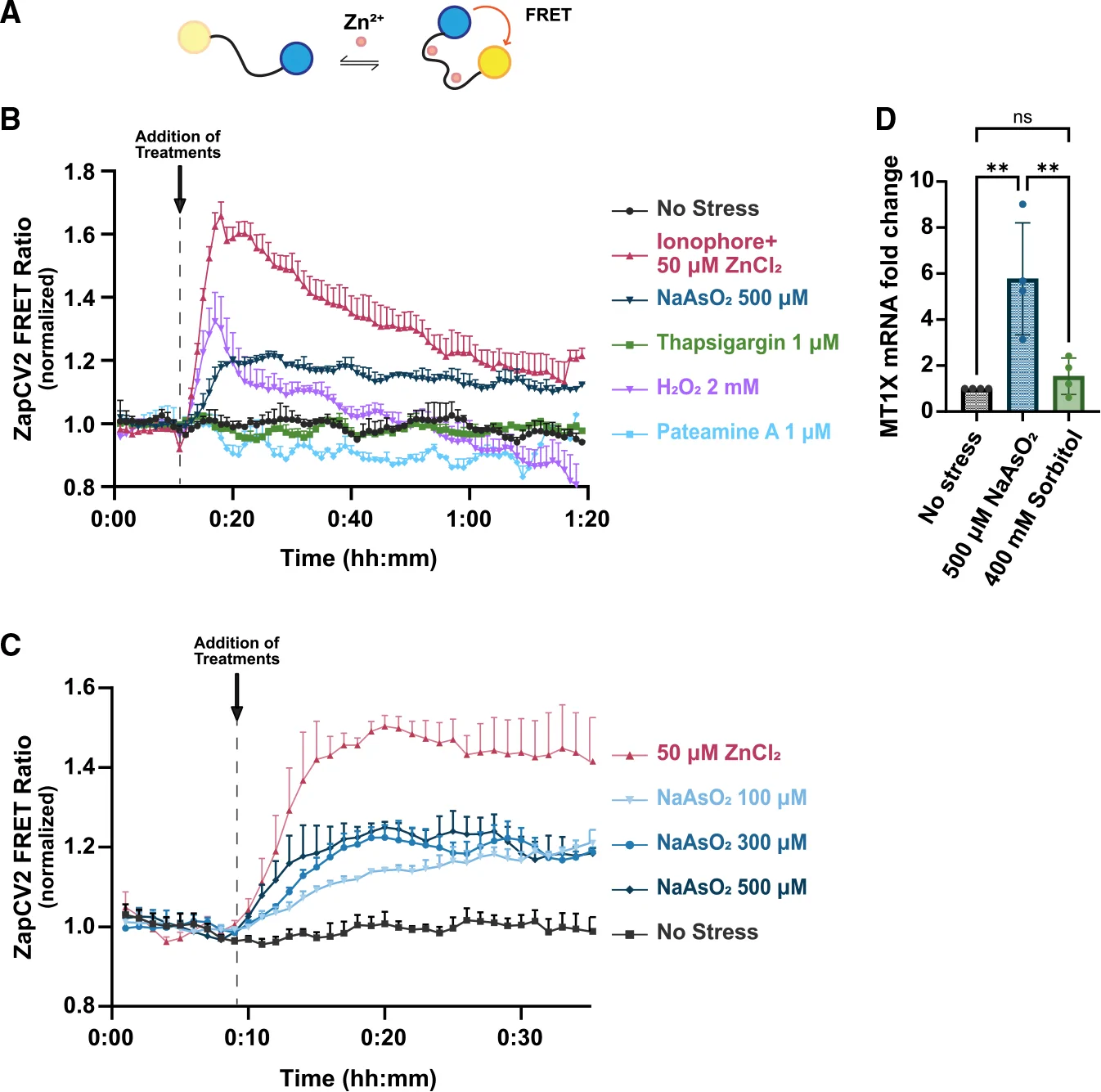
Metallothioneins are upregulated during oxidative stress due to increase in zinc concentration (A) Diagram of the ZapCV2 zinc-binding FRET sensor. (B) Cytosolic Zn^2+^ levels in response to extracellular perturbations in U-2 OS cells expressing the ZapCV2 sensor. The cells were subjected to 500 μM sodium arsenite stress, 2 mM hydrogen peroxide, 1 μM thapsigargin, 1 μM of the translation arrestor pateamine A, or 50 μM zinc + ionophore. The FRET ratio was normalized by dividing the FRET value of each frame by the mean FRET ratio of the 15 baseline frames immediately prior to the addition of the first treatment. Traces combine the averages of 2 (pateamine A) or 3 independent experiments (all others), each with a minimum of 18 individual cells quantified. Data are represented as the mean ± SEM. (C) Cytosolic Zn^2+^ levels in response to a dose curve of sodium arsenite between 100 and 500 μM. Traces combine the averages of 3 independent experiments, each with a minimum of 40 individual cells quantified. Data are represented as the mean ± SEM. (D) RT-qPCR analysis of MT1X gene under unstressed or stressed conditions. GAPDH was used to normalize the samples. Mean ± SD values are based on a minimum of 4 independent experiments. Individual experiments were done in triplicate. Data were analyzed with one-way ANOVA, corrected with Tukey’s multiple-comparisons test. ns, non-significant, *p* > 0.05; *, *p* ≤ 0.05; **, *p* ≤ 0.01.

**Figure 2.**
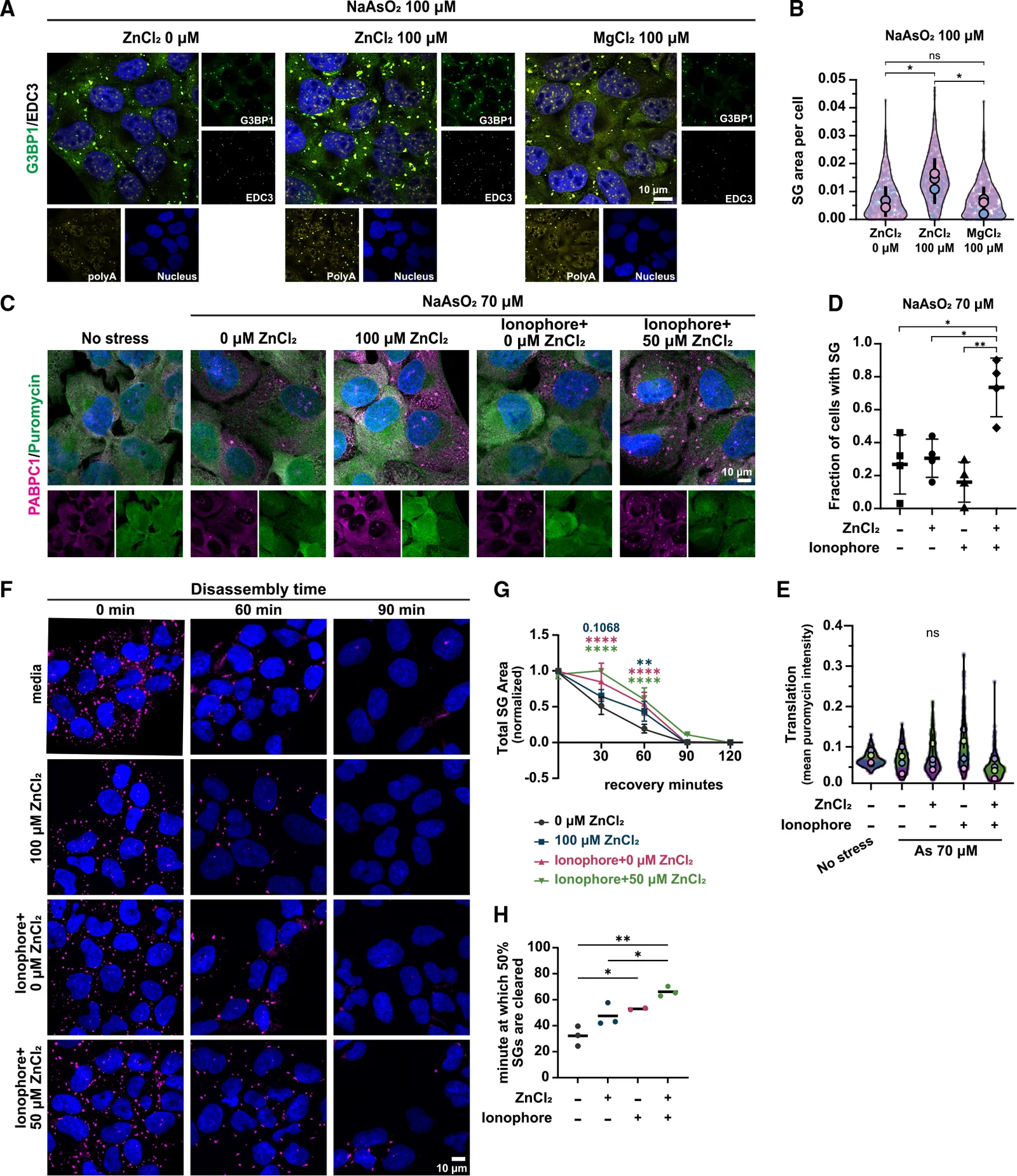
Zinc leads to increased stress granule formation (A) Representative images of immunofluorescence for G3BP1 (green), EDC3 (gray), polyA FISH (yellow), and DAPI stain (blue) in U-2 OS cells subjected to 100 μM sodium arsenite (NaAsO2) (i) and 100 μM ZnCl_2_ (ii) or 100 μM MgCl_2_ (iii). (B) Quantification of the total stress granule area per cell normalized to the cell cytoplasm area. Replicate mean ± SD values are denoted as large dots. Averages from 3 independent experiments with a minimum of 50 cells per condition were analyzed with one-way ANOVA, corrected with Tukey’s multiple-comparisons test. (C) Representative images of immunofluorescence for PABPC1 (magenta), puromycin (green), and DAPI stain (blue) in U-2 OS cells subjected to 70 μM sodium arsenite (ii) and 100 μM ZnCl_2_ (iii), and ionophore alone (iv) or ionophore with 50 μM ZnCl_2_ (v). (D) Fraction of the analyzed cell population presenting at least one stress granule after treatment with 70 μM sodium arsenite for 60 min. Data are represented as mean ± SD. Data were analyzed with one-way ANOVA, corrected with Tukey’s multiple-comparisons test. Four biological replicates, each with an average of 83 cells, were analyzed per condition. The cells treated with zinc received 100 μM ZnCl_2_. The cells treated with zinc ionophore received 50 μM ZnCl_2_ + ionophore. (E) Quantification of puromycin staining represented as the mean intensity per cell (arbitrary units). Replicate mean ± SD values are denoted as large dots. Averages from 4 independent experiments were analyzed with one-way ANOVA, corrected with Tukey’s multiple-comparisons test. Replicates with less than 10 positive cells in at least one condition were excluded from this quantification. The cells treated with zinc received 100 μM ZnCl_2_. The cells treated with zinc ionophore received 50 μM ZnCl_2_ + ionophore. (F) Time course of SG disassembly in U-2 OS cells. The cells were pre-exposed to 500 μM sodium arsenite for 30 min, washed twice, and allowed to recover in growth media or growth media supplemented with 100 μM ZnCl_2_ or 50 μM zinc + ionophore. 0 min denotes the time point at which stress was removed. Immunofluorescence for PABPC1. (G) SG disassembly quantified as total SG area over time, with each dataset normalized to the SG area at t = 0 min and represented as mean ± SD. Three independent experiments were analyzed with two-way ANOVA and Tukey’s multiple-comparisons test. Significance relative to the 0 μM ZnCl2 control is indicated. (H) The estimated time at which half of the stress granule area was cleared was interpolated from a spline curve of each dataset and analyzed with one-way ANOVA, corrected with Tukey’s multiple-comparisons test. ns, non-significant, *p* > 0.05; *, *p* ≤ 0.05; **, *p* ≤ 0.01; ***, *p* ≤ 0.001; ****, *p* ≤ 0.0001. Scale bars, 10 μm, unless otherwise specified.

**Figure 3.**
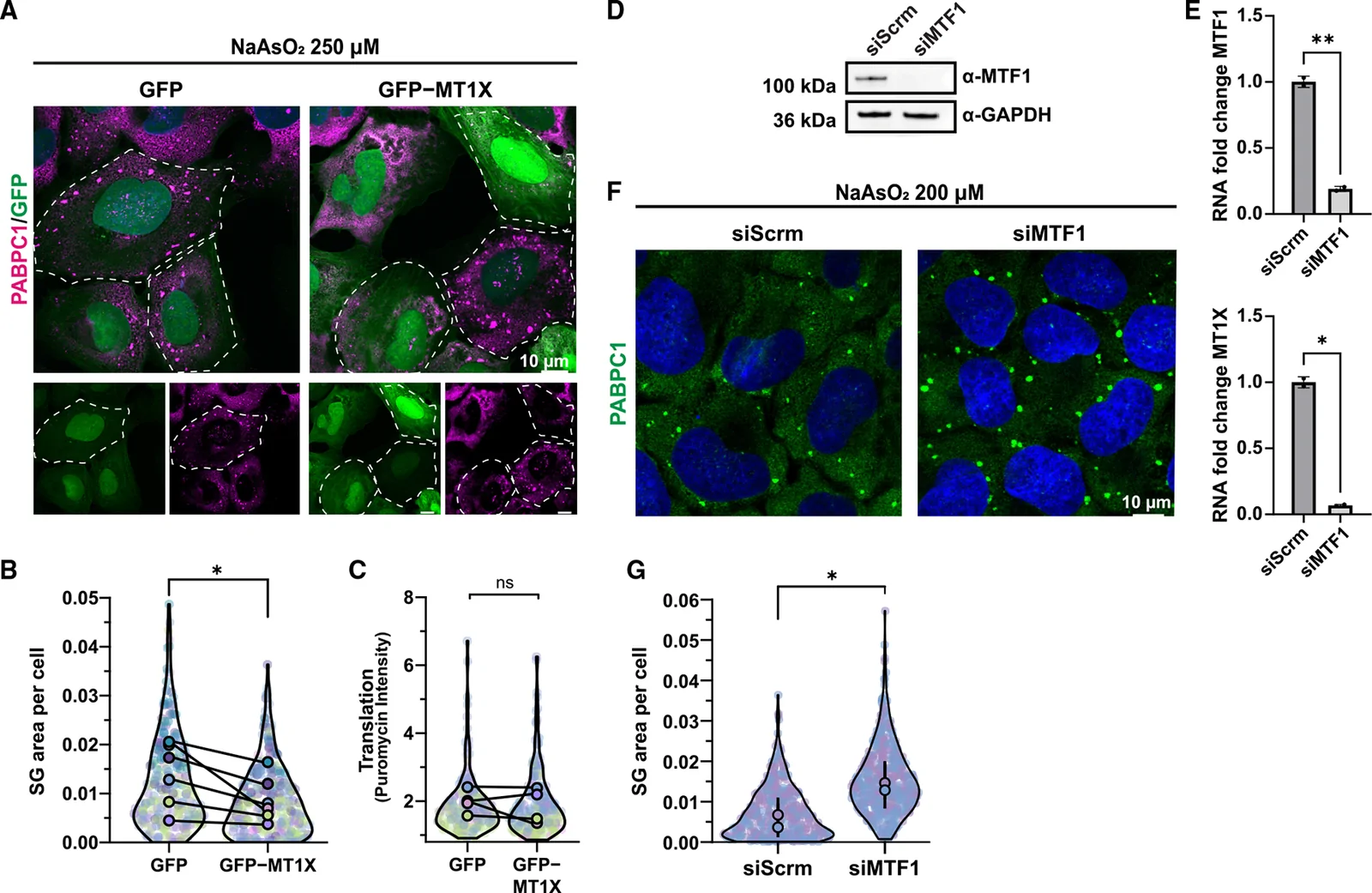
Metallothioneins limit stress granule formation (A) Immunofluorescence of U-2 OS cells constitutively expressing GFP or GFP-MT1X and exposed to 250 μM of sodium arsenite for 1 h. Magenta, PABPC1; green, GFP. White dashed lines demark individual cells. (B) Quantification of the total stress granule area normalized to the cell’s cytoplasm area. Cells with outlier intensities of GFP (>0.025 and <0.13 arbitrary intensity units) were excluded from this analysis so that the mean GFP expression levels between the cell lines were even within replicates. Replicate means are denoted as large dots. Averages from 6 independent experiments with a minimum of 15 cells per condition were analyzed with paired, two-tailed *t* tests. (C) Quantification of puromycin staining of the subpopulation of (B), represented as the mean intensity per cell (arbitrary units). Replicate means are denoted as large dots. Averages from 4 independent experiments with a minimum of 15 cells per condition were analyzed with paired, two-tailed *t* tests. (D) Immunoblot of MTF1 knockdown, with GAPDH as a loading control. Representative of three independent experiments. (E) RT-qPCR analysis of MTF1 (top) and MT1X (bottom) in siScramble control cells and siMTF1 cells. GAPDH was used to normalize the samples. Mean ± SD values are based on 2 independent experiments. Individual experiments were performed in triplicate. Data were analyzed with Welch’s *t* test. (F) Representative images of immunofluorescence for PABPC1 (green) in U-2 OS siScramble control and MTF1-Kd cells exposed to 200 μM sodium arsenite for 1 h. (G) Quantification of the total stress granule area per cell normalized to the cell cytoplasm area. Replicate mean ± SD values are denoted as large dots. Averages from 2 independent experiments with 150 cells per condition were analyzed with Welch’s *t* test. ns, non-significant, *p* > 0.05; *, *p* ≤ 0.05; **, *p* ≤ 0.01; ***, *p* ≤ 0.001; ****, *p* ≤ 0.0001. Scale bar, 10 μm, unless otherwise specified.

**Figure 4.**
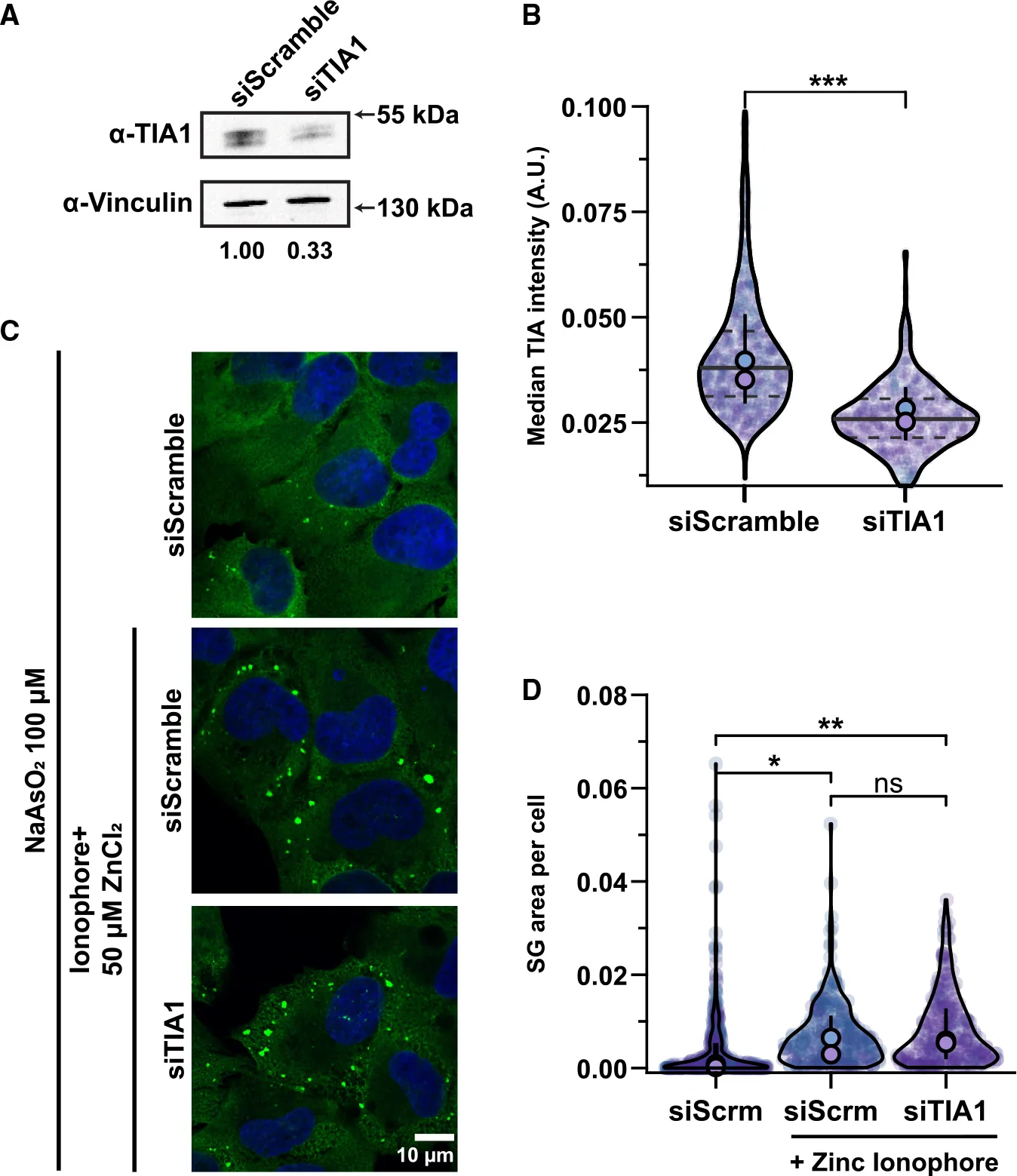
Zinc’s increase of stress granule is not due to TIA1 (A) Immunoblot of TIA1 knockdown, with vinculin as a loading control. Representative of two independent replicates. (B) Quantification of immunofluorescence of TIA1 as median intensity (arbitrary units). Replicate means are denoted as large dots. From each replicate, *n* = 20 cells were randomly sampled and analyzed with Welch’s two-tailed *t* test. (C) Immunofluorescence of PABPC1 in U-2 OS cells treated with scrambled siRNA or TIA1 siRNA and subjected to arsenite stress with or without 50 μM zinc + ionophore. PABPC1, green; nuclear stain, in blue. (D) Quantification of the total stress granule area per cell normalized to the cell cytoplasm area. Replicate means are denoted as large dots. From each replicate, *n* = 20 cells were randomly sampled and analyzed with Welch’s two-tailed *t* test. ns, non-significant, *p* > 0.05; *, *p* ≤ 0.05; **, *p* ≤ 0.01; ***, *p* ≤ 0.001; ****, *p* ≤ 0.0001. Scale bars, 10 μm, unless otherwise specified.

**Figure 5.**
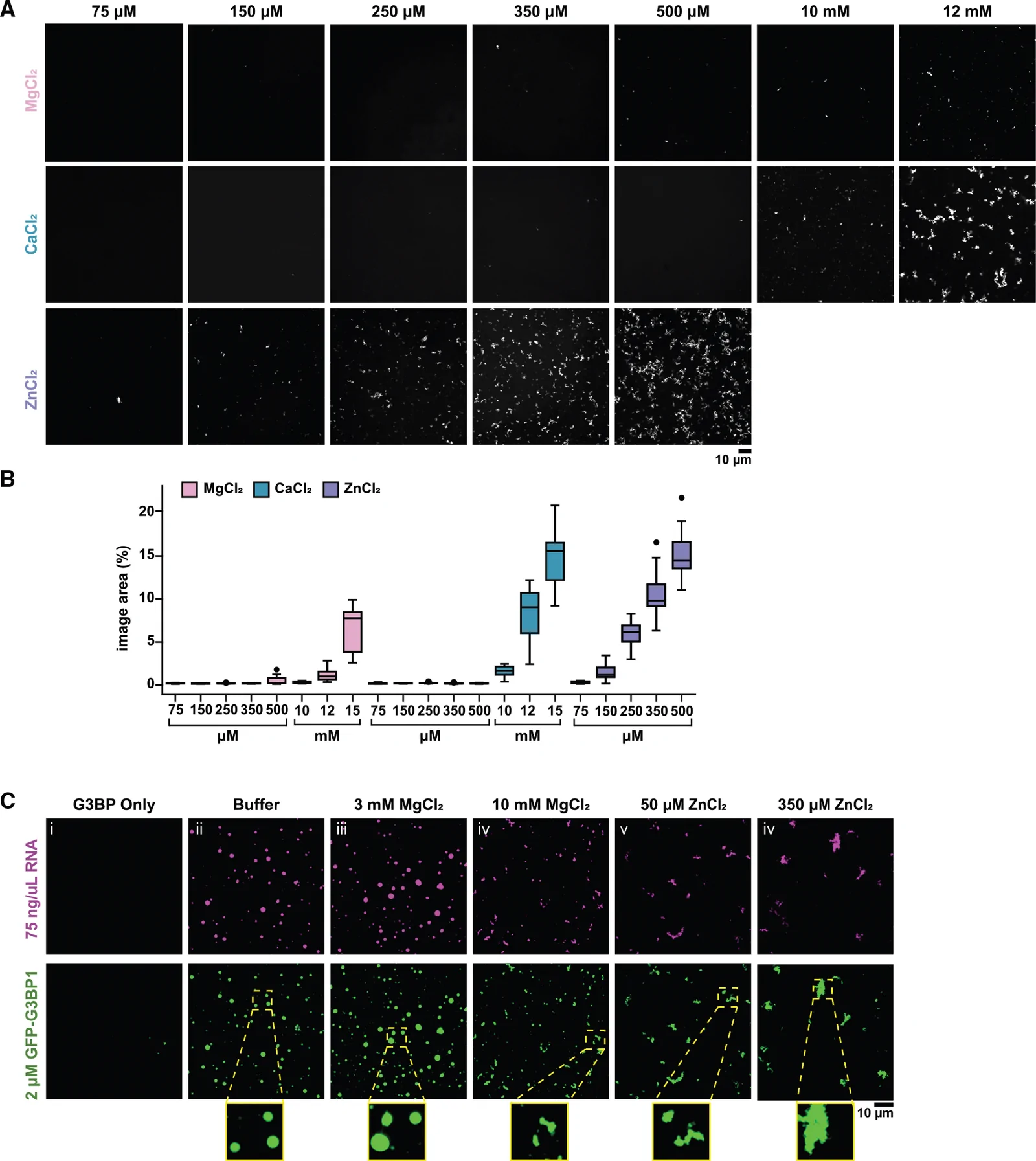
Zinc is a potent RNA condenser *in vitro* (A) 150 ng/μL Cy3-luciferase condensates in the presence of increasing concentrations of MgCl_2_, CaCl_2_, or ZnCl_2_ in the condensation buffer (20 mM HEPES pH 7.5, 10% PEG, and 150 mM NaCl) imaged after 1 h incubation at 25°C. (B) RNA condensates were masked, and size was quantified as fraction of the area of the micrograph. A minimum of 15 images across 3 independent experiments performed in (A) were analyzed. All images were background corrected to the buffer control. (C) GFP-G3BP1-RNA co-condensates were formed using 2 μM G3BP1 and 75 ng/μL Cy3-labeled luciferase RNA in condensation buffer supplemented with 3 mM MgCl_2_ (iii), 10 mM MgCl_2_ (iv), or 50 μM ZnCl_2_ (v). Scale bars, 10 μm, unless otherwise specified.

**Figure 6.**
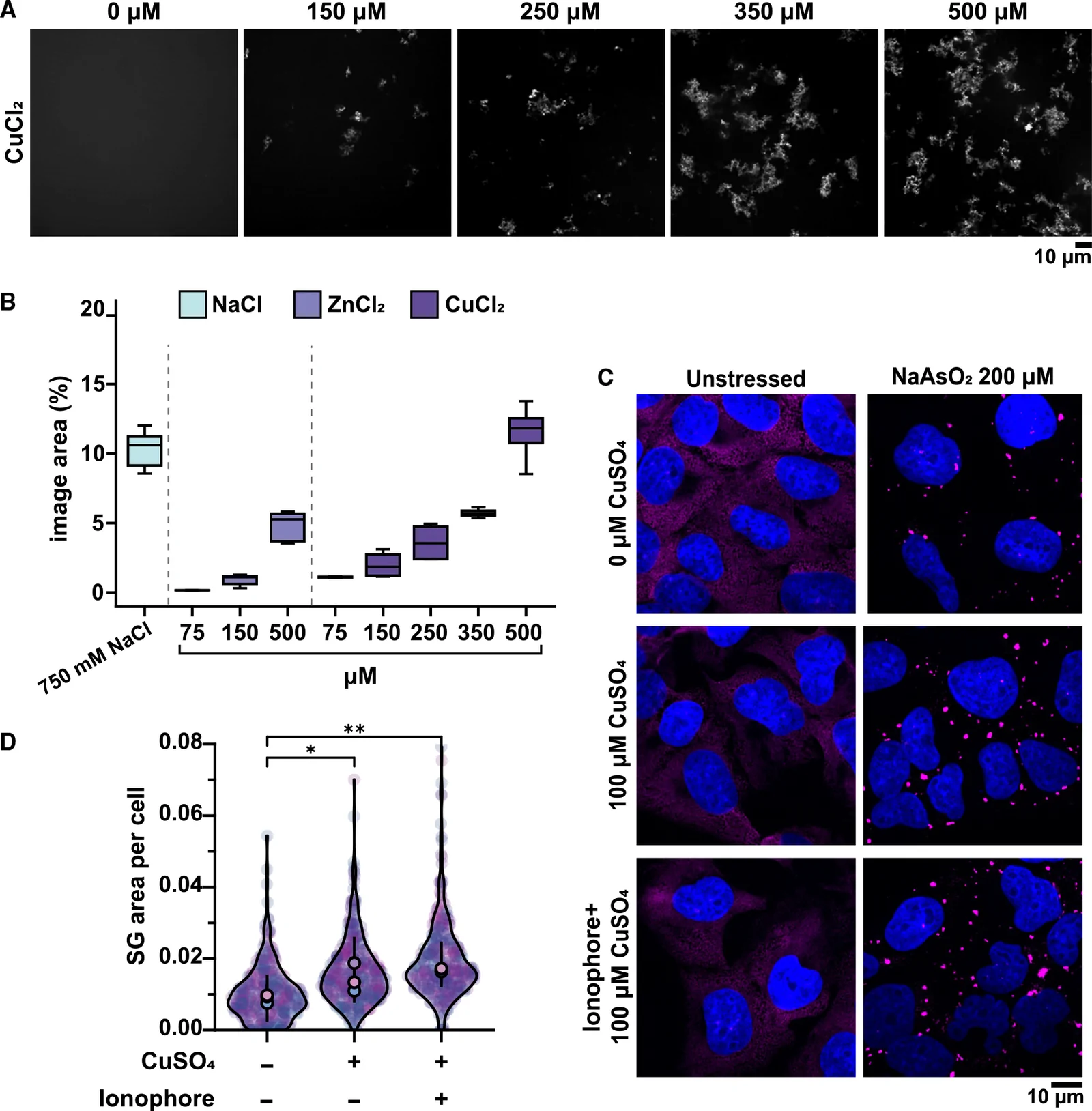
Copper replicates the effects of zinc *in vitro* and in cells (A) 150 ng/μL Cy3-luciferase condensates in the presence of CuCl_2_ or ZnCl_2_ in condensation buffer (20 mM HEPES pH 7.5, 10% PEG, and 150 mM NaCl) imaged after 45 min incubation at 25°C. (B) Quantification of RNA condensates made with ZnCl_2_ or CuCl_2_ between the concentrations of 75 and 500 μM. RNA condensates were masked, and size was quantified as a fraction of the area of the micrograph. A minimum of 15 images across 3 independent experiments performed in (A) were analyzed. (C) Representative images of immunofluorescence for PABPC1 (magenta) and DAPI stain (blue) in U-2 OS cells subjected to no stress or 200 μM sodium arsenite and 0 or 100 μM of copper sulfate or ionophore + 50 μM copper sulfate for 1 h. (D) Quantification of the total stress granule area per cell normalized to the cell cytoplasm area in (C). Replicate mean ± SD values are denoted as large dots. Averages from 4 independent experiments were analyzed with one-way ANOVA, corrected with Tukey’s multiple-comparisons test. ns, non-significant, *p* > 0.05; *, *p* ≤ 0.05; **, *p* ≤ 0.01. Scale bars, 10 μm, unless otherwise specified.

**Figure 7.**
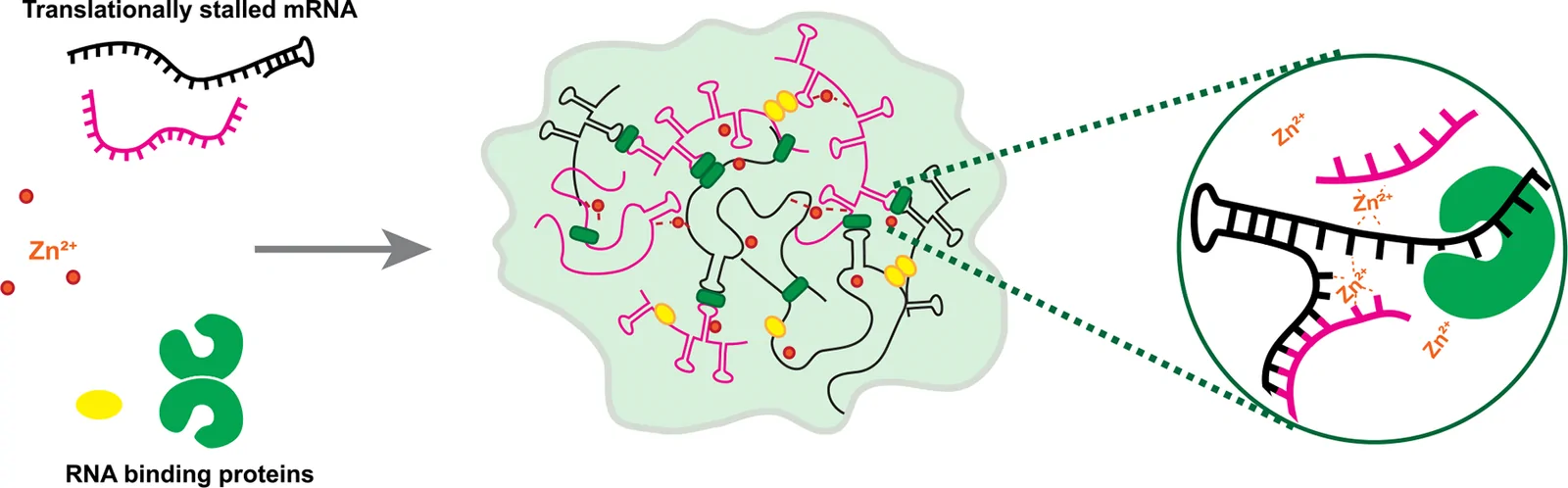
Zinc aids in stress granule formation through condensation of RNA

**KEY RESOURCES TABLE:** 

REAGENT or RESOURCE	SOURCE	IDENTIFIER

Antibodies		

Mouse monoclonal anti-G3BP1	Abcam	Cat#ab56574; RRID:AB_941699
Rabbit polyclonal anti-PABPC1	Abcam	Cat# ab21060; RRID:AB_777008
Rabbit anti-H3 antibody HRP conjugate	Cell Signaling Technology	Cat# 12648; RRID:AB_2797978
Rabbit polyclonal anti-GFP	Thermo Fisher Scientific	Cat#A-11122; RRID:AB_221569
Mouse monoclonal anti-puromycin cl 12D10	Sigma-Aldrich	Cat#MABE343; RRID:AB_2566826
Rabbit polyclonal anti-TIA1	ProteinTech	Cat#12133-2-AP; RRID:AB_2201427
Rabbit monoclonal anti-phospho S51 eIF2S1	Abcam	Cat#ab32157; RRID:AB_732117
Rabbit polyclonal anti-eIF2 alpha	Cell Signaling Technology	Cat#9722S; RRID:AB_2230924
Rabbit polyclonal anti-MTF1	ProteinTech	Cat#25383-1-AP; RRID:AB_2880053
Mouse monoclonal anti-Vinculin	Sigma-Aldrich	Cat#V9131; RRID:AB_477629
Goat anti-mouse IgG Alexa Fluor 488 conjugate secondary antibody	Abcam	Cat#ab150113; RRID:AB_2576208
Goat anti-rabbit IgG Alexa Fluor 647 conjugate secondary antibody	Abcam	Cat#ab150077; RRID:AB_2630356
Anti-mouse IgG, HRP-linked antibody	Cell Signaling Technology	Cat#7076S; RRID:AB_330924
Anti-rabbit IgG, HRP-linked antibody	Cell Signaling Technology	Cat#7074S; RRID:AB_2099233

Bacterial and virus strains		

E. coli HST08 Stellar^™^	TakaraBio	Cat# 636763
Lenti-EF1-Blast_GFP	This work	N/A
Lenti-EF1-Blast_GFP-MT1X	This work	N/A

Chemicals, peptides, and recombinant proteins		

Sodium (meta)arsenite >90%	Sigma-Aldrich	Cat# S7400
Pateamine A (PatA)	Low et al.^20^	N/A
Puromycin Dihydrochloride (Puro)	Gibco	Cat# A1113803
TRIzol Reagent	Thermo Fisher Scientific	Cat#15596018
Lipofectamine 3000 Transfection Reagent	Thermo Fisher Scientific	Cat#L3000008
Lipofectamine RNAiMAX Transfection Reagent	Thermo Fisher Scientific	Cat#13778075
Lipofectamine 2000 Transfection Reagent	Thermo Fisher Scientific	Cat#11668027
Zinc chloride	Sigma-Aldrich	Cat#Z0152-50G
Magnesium chloride hexahydrate	Sigma-Aldrich	Cat#7791-18-6
Calcium chloride dihydrate	Sigma-Aldrich	Cat#C3881-500G
5M NaCl	Thermo Fisher Scientific	Cat#Am9760G
Poly(ethylene glucol) 3350	Sigma-Aldrich	Cat#88276-1kg-F
HEPES	Thermo Fisher Scientific	Cat#BP310-500
XhoI	New England Biolabs	Cat#R0146L
XbaI	New England Biolabs	Cat#R0145L
AfeI	New England Biolabs	Cat#R0652L
Cyanine 3-UTP (enhanced)	Enzo Life Sciences	Cat#ENZ-42505
Ampicillin	Sigma-Aldrich	Cat#A9518
cOmplete^™^ Protease Inhibitor Cocktail, EDTA-Free, Mini, Tablets	Sigma-Aldrich	Cat#6538282001
Phosstop^™^-phosphatase inhibitor	Sigma-Aldrich	Cat#4906837001
Triton X-100	Thermo Fisher Scientific	Cat#AC327371000
TURBO DNase	Thermo Fisher Scientific	Cat#AM1907
Pierce RIPA Buffer	Thermo Fisher Scientific	Cat#89900
Paraformaldehyde 4% solution	Santa Cruz Biotechnology	Cat#30525-89-4
Tween 20	Sigma-Aldrich	Cat#P9416
Prolong Glass Antifade Mountant	Thermo Fisher Scientific	Cat#P36982
D-(+)-Glucose solution	Sigma-Aldrich	Cat#G8644
2-Mercaptopyridine N-oxide 99% (Pyrithione)	Sigma-Aldrich	Cat#188549
Saponin	Sigma-Aldrich	Cat#47036
Tris(2-pyridylmethyl) amine 98% (TPA)	Sigma-Aldrich	Cat#723134
Chelex-100, sodium free	Bio-Rad	Cat#142-1253
Hoechst 3342	Thermo Fisher Scientific	Cat#62249
DAPI	Thermo Fisher Scientific	Cat#62248
G418 sulfate	Thermo Fisher Scientific	Cat#10131035
Blotting-Grade Blocker nonfat dry milk	Bio-Rad	Cat#1706404
Bovine Serum Albumin	Sigma-Aldrich	Cat#A9647-100G
NuPAGE^™^ MES SDS Running Buffer (20X)	Thermo Fisher Scientific	Cat#NP0002
Proteinase K	Thermo Fisher Scientific	Cat#EO0491
EDTA (Nuclease Free)	Thermo Fisher Scientific	Cat#AM9260G
Poly-L-lysine	Santa Cruz Biotechnology	Cat#sc-286689A
Blasticidin S hydrochloride	Sigma-Aldrich	Cat#15205
Polybrene	Millipore	Cat#TR-1003-G

Critical commercial assays		

MEGAscript T7 Transcription Kit	Invitrogen	Cat#AM1334
MEGAclear Transcription Clean-up Kit	Invitrogen	Cat#AM1908
Zymo Direct-zol Miniprep Kit	Zymo Research	Cat#R2051
Clarity Western ECL Substrate	Bio-Rad	Cat#1705061
SuperScript IV First-Strand Synthesis System	Invitrogen	Cat#18091050
In-Fusion Snap Assembly Master Mix	TakaraBio	Cat#638947
LightCycler 480 SYBR Green I Master mix	Roche Life Science	Cat#4707516001
ZymoPURE Plasmid Miniprep Kit	Zymo Research	Cat#D4212
Whole Plasmid Sequencing	Plasmidsaurus	N/A

Experimental models: Cell lines		

U-2 OS cells	Kedersha et al.^4^	N/A
A549 cells	ATCC	Cat#CCL-185
HEK293T cells	ATCC	Cat#CRL-3216
U-2 OS-GFP	This work	N/A
U-2 OS-GFP-MT1X	This work	N/A

Oligonucleotides		

See Table S1 for list of primers and oligonucleotides	N/A	N/A
Silencer^™^ Select Negative Control No. 1 siRNA	Thermo Fisher Scientific	Cat#4390843
Silencer^™^ Select Pre-Designed siRNA TIA1 (#s14131)	Thermo Fisher Scientific	Cat#4392420/#s14131
Silencer^™^ Select Pre-Designed siRNA MTF1 (#s9027)	Thermo Fisher Scientific	Cat#4392420/#s9027
Silencer^™^ Select Pre-Designed siRNA MTF1 (#s9028)	Thermo Fisher Scientific	Cat#4392420/#s9028
Silencer^™^ Select Pre-Designed siRNA MTF1 (#s9029)	Thermo Fisher Scientific	Cat#4392420/#s9029

Recombinant DNA		

GST-EGFP-G3BP1	Yang et al.^10^	N/A
T7 luciferase	Promega	Cat#L4741
PB-NES-ZapCV2	Fiedler et al.,^35^ Addgene	Cat#222950; RRID:Addgene_222950
pTwist-CMV_eGFP-MT1X	This work, Twist Bioscience	N/A

Software and algorithms		

ImageJ (FIJI)	NIH	https://imagej.nih.gov/ij/; RRID:SCR_003070
Prism 10	GraphPad	https://www.graphpad.com/features; RRID:SCR_002798
CellProfiler Image Analysis Software Version 4.2.5	CellProfiler	https://cellprofiler.org/; RRID:SCR_007358
MATLAB R2025a	Mathworks	https://www.mathworks.com/products/matlab.html; RRID:SCR_001622
R 4.5.1	CRAN	https://cran.r-project.org/
R studio	Posit	https://posit.co/products/open-source/rstudio/

Other		

iBlot 2 Gel Transfer Device	Invitrogen	Cat# IB21001
iBright Imaging System	Thermo Fisher Scientific	Cat# A44115
Nupage^™^ 4–12% Bis-Tris Protein Gels, 1.0 mm, 12-well	Thermo Fisher Scientific	NP0335BOX
iBlot^™^ 2 nitrocellulose transfer stack	Invitrogen	Cat# IB23001
60 u-Dish 35 mm	Ibidi	Cat# 81156
96 well glass bottom plate #1.5 cover glass	Cellvis	Cat# P96.1.5H-N
12 mm # 1.5 round glass cover slips	Warner Instruments	Cat# cs-12r15

## Highlights

- Oxidative stress causes an increase in free zinc in the cell cytoplasm
- Increased zinc increases stress granule formation without affecting translation
- Reduction of endogenous free zinc decreases stress granule formation
- Zinc directly facilitates RNA condensation at 100× lower concentrations than magnesium
