# Supplementary material for: Zinc increases stress granule formation by promoting RNA condensation
Source: Cell Rep. Author manuscript; Available in PMC 2026 Oct 14. (PMC13663885; doi:10.1016/j.celrep.2026.117870)
Supplement: Supplementary file 1 [file NIHMS2212219-supplement-1.pdf]

Cell Reports, Volume *45*

Supplemental information

Zinc increases stress granule formation by promoting RNA condensation

Luisa Macedo de Vasconcelos, Nina Ripin, and Roy Parker

Figure S1

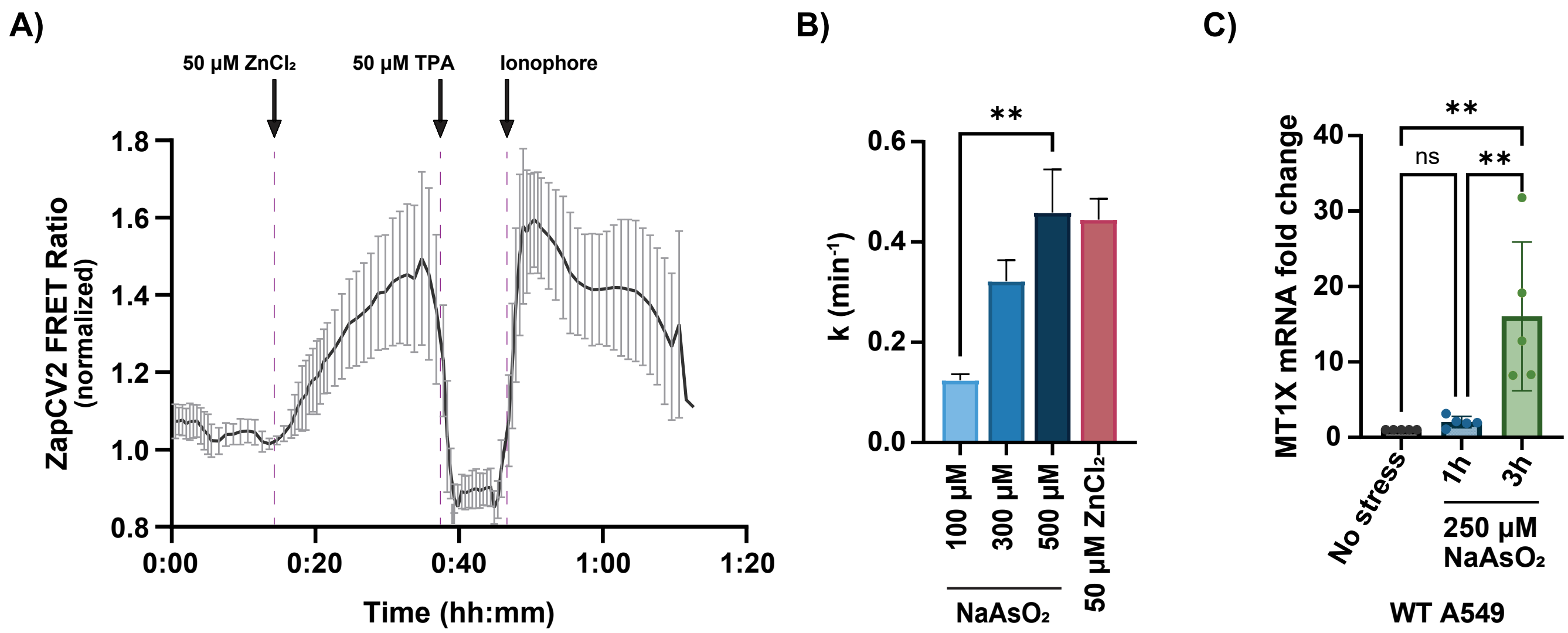

A)
50 µM ZnCl₂
50 µM TPA
Ionophore
ZapCV2 FRET Ratio
(normalized)
1.8
1.6
1.4
1.2
1.0
0.8
0:00
0:20
0:40
1:00
1:20
Time (hh:mm)
B)
**
k (min-1)
0.6
0.4
0.2
0.0
100 µM
300 µM
500 µM
50 µM ZnCl₂
NaAsO₂
C)
**
ns
**
MT1X mRNA fold change
40
30
20
10
0
No stress
1h
3h
250 µM
NaAsO₂
WT A549

Figure S1: ZapCV2 reporter is successful in tracking free zinc changes in live cells, and metallothionein upregulation is not specific to cell type, related to Figure 1

(A) Calibration of ZapCV2 sensor in U-2 OS cells. TPA is used to establish the minimal FRET ratio and zinc ionophore is used to determine FRET ration at saturation. The FRET ratio was normalized by dividing the FRET value of each frame with the mean FRET ratio of the five frames immediately prior to the addition of the first treatment. Traces combine the averages of 4 independent experiments each with a minimum of 40 individual cells quantified. Data represented as mean ± sem.

(B) Rate constants obtained from a Gompertz non-linear fit (Figure 1 D). Only data points between t = 10 and t = 35 min were fit. Data represented as mean ± sem.

(C) Reverse transcription-qPCR analysis of metallothionein 1X gene under unstressed or stressed conditions in A549 cells. GAPDH was used to normalize the samples. Mean ± sd values are based on a minimum of 4 independent experiments. Individual experiments done in triplicate. Data analyzed with one-way ANOVA, corrected with Tukey's multiple comparisons test.

ns = non-significant, $p > 0.05$; * = $p \leq 0.05$; ** $p \leq 0.01$

Figure S2

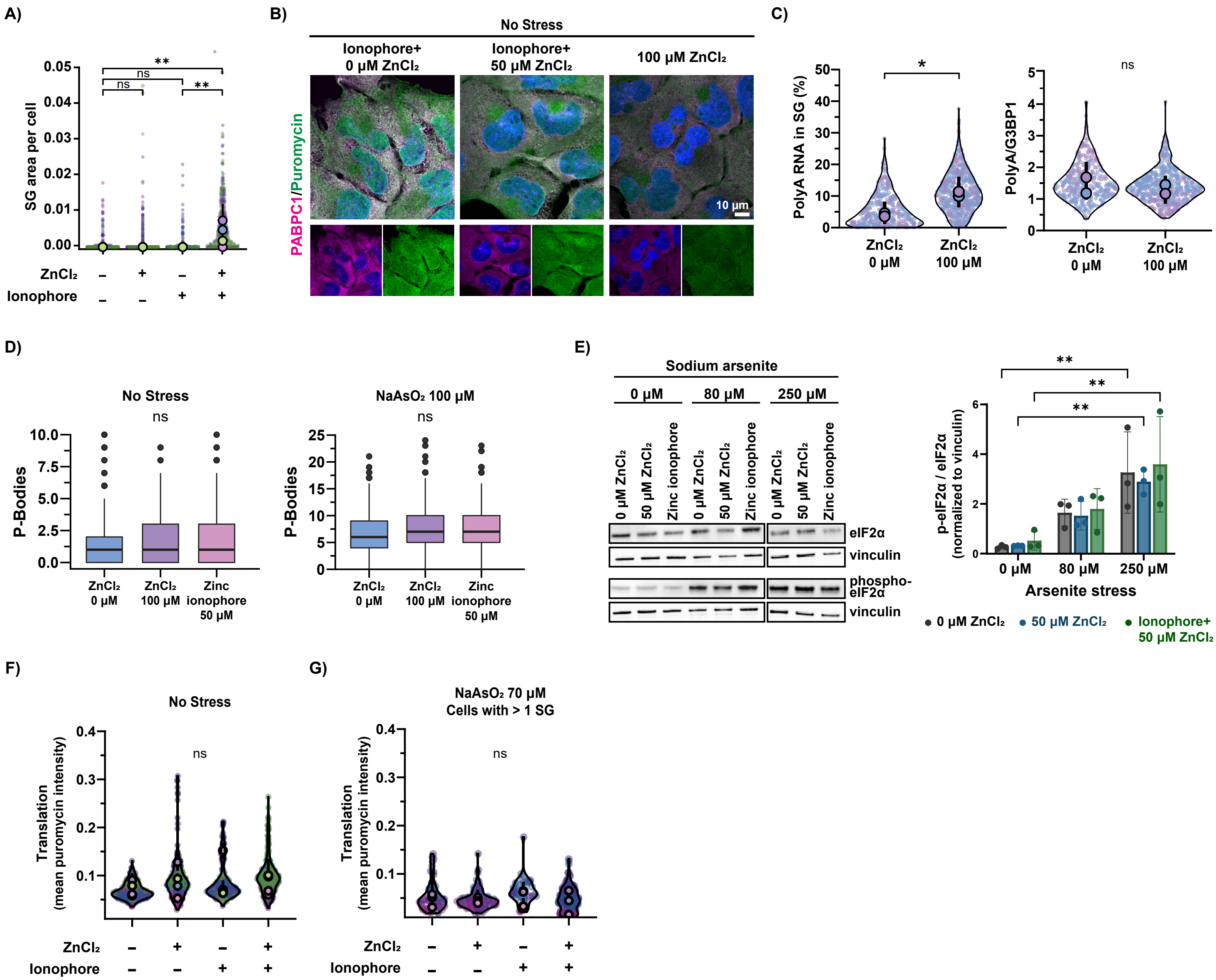
A)
SG area per cell
ZnCl₂
Ionophore
B)
No Stress
Ionophore+ 0 µM ZnCl₂
Ionophore+ 50 µM ZnCl₂
100 µM ZnCl₂
PABPC1/Puromycin
10 µm
C)
PolyA RNA in SG (%)
PolyA/G3BP1
ZnCl₂ 0 µM
ZnCl₂ 100 µM
D)
No Stress
NaAsO₂ 100 µM
P-Bodies
ZnCl₂ 0 µM
ZnCl₂ 100 µM
Zinc ionophore 50 µM
E)
Sodium arsenite
0 µM
80 µM
250 µM
0 µM ZnCl₂
50 µM ZnCl₂
Zinc ionophore
eIF2α
vinculin
phospho-eIF2α
p-eIF2α / eIF2α (normalized to vinculin)
Arsenite stress
0 µM ZnCl₂
50 µM ZnCl₂
Ionophore+ 50 µM ZnCl₂
F)
No Stress
Translation (mean puromycin intensity)
G)
NaAsO₂ 70 µM
Cells with > 1 SG
ZnCl₂
Ionophore

Figure S2: Zinc leads to increased stress granule formation without changes to translational repression, related to Figure 2

(A) Quantification of total stress granule area per cell normalized to cell cytoplasm area. Cells treated with zinc received 100 μM $ZnCl_2$. Cells treated with zinc ionophore received 50 μM $ZnCl_2$ + ionophore. Replicate means ± sd are denoted as large dots. Averages from 4 independent experiments with a minimum of 49 cells per condition were analyzed with one-way ANOVA, corrected with Tukey's multiple comparisons test.

(B) Representative images of immunofluorescence for PABPC1 (magenta), puromycin (green) and DAPI stain (blue) in unstressed U-2 O S cells treated ionophore (i), ionophore with 50 μM $ZnCl_2$ (ii) or 100 μM $ZnCl_2$ (iii).

(C) The percentage of integrated mRNA FISH signal in stress granules (left) and the ratio of integrated stress granule intensities of mRNA FISH and G3BP1 (right) in cells stressed with 100 μM sodium arsenite with and without 100 μM $ZnCl_2$ Averages from 2 independent experiments with a minimum of 50 cells per condition were analyzed with Welch's two-tailed t-test.

(D) P-body count in unstressed (left) and 100 μM sodium arsenite treated (right) with no added zinc, 100 μM $ZnCl_2$ or 50 μM $ZnCl_2$ + ionophore. Averages from 3 independent experiments with a minimum of 50 cells per condition were analyzed with one-way ANOVA, corrected with Tukey's multiple comparisons test.

(E) Immunoblot for eIF2α and phosphorylated eIF2α with vinculin as a loading control. (Left) Representative of three independent experiments. Densiometric analysis of eIF2α phosphorylation (Right). Blot bands were normalized to the loading control, and values were calculated as the ratio of phosphorylated to total eIF2α. Analysis was done with two-way ANOVA corrected with Tukey's multiple comparisons test. Individual blots are separated by black boxes.

(F) Quantification of puromycin staining represented as mean intensity per cell (arbitrary units) for unstressed cells. Replicate means + sd are denoted as large dots. Averages from 4 independent experiments were analyzed with one-way ANOVA, corrected with Tukey's multiple comparisons test. Cells treated with zinc received 100 μM $ZnCl_2$. Cells treated with zinc ionophore received 50 μM $ZnCl_2$ + ionophore.

(G) Quantification puromycin staining represented as mean intensity per cell of the population of cells that presents at least one stress granule. Replicate means + sd are denoted as large dots. Averages from 3 independent experiments were analyzed with one-way ANOVA, corrected with Tukey's multiple comparisons test. Cells treated with zinc received 100 μM $ZnCl_2$. Cells treated with zinc ionophore received 50 μM $ZnCl_2$ + ionophore.

ns = non-significant, $p > 0.05$; * = $p \le 0.05$; ** $p \le 0.01$; *** $p \le 0.001$; **** $p \le 0.0001$. Scale bar, 10 μm unless otherwise specified.

Figure S3

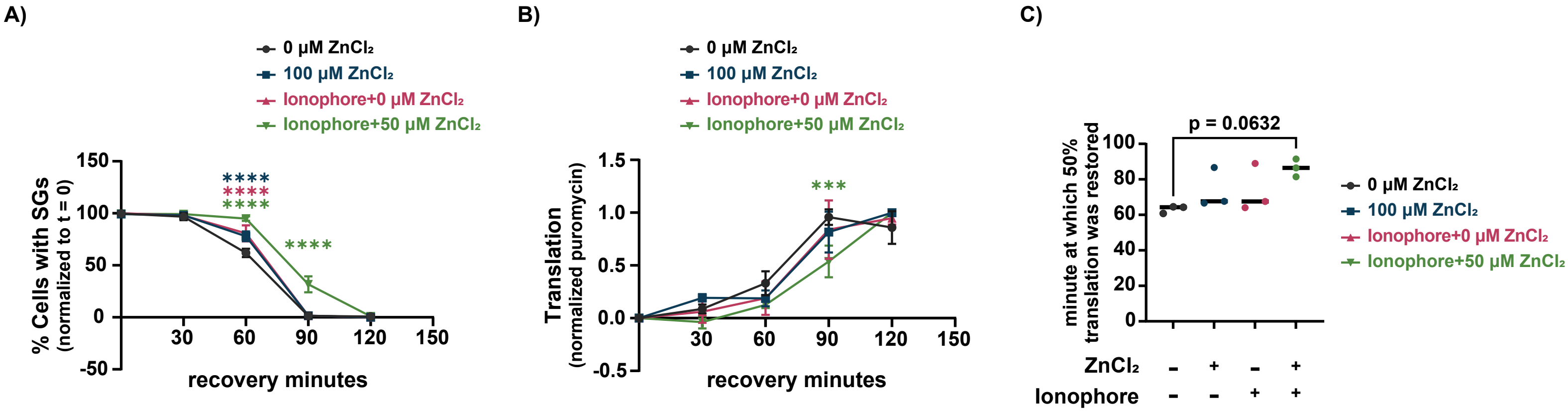

A)
0 µM ZnCl₂
100 µM ZnCl₂
Ionophore+0 µM ZnCl₂
Ionophore+50 µM ZnCl₂
% Cells with SGs
(normalized to t = 0)
recovery minutes
****
****
****
****
B)
Translation
(normalized puromycin)
recovery minutes
***
C)
minute at which 50%
translation was restored
p = 0.0632
ZnCl₂
Ionophore

Figure S3: Zinc delays stress granule clearance, related to Figure 2

(A) Percentage of cells with at least 1 stress granule at timepoints t = 0, 30, 60, 90 and 120 minutes, normalized to t = 0 min. Three independent experiments were analyzed with two-way ANOVA and Tukey's multiple comparisons test. Cells treated with zinc received 100 μM $ZnCl_2$. Cells treated with zinc ionophore received 50 μM $ZnCl_2$ + ionophore.

(B) Puromycin incorporation during stress granule disassembly. Each condition is normalized to the highest mean puromycin intensity of that condition and represented as mean ± sd. Three independent experiments were analyzed with two-way ANOVA and Tukey's multiple comparisons test. Cells treated with zinc received 100 μM $ZnCl_2$. Cells treated with zinc ionophore received 50 μM $ZnCl_2$ + ionophore.

(C) The estimated time at which 50% of translation was restored was interpolated from a spline curve of each dataset and were analyzed with one-way ANOVA, corrected with Tukey's multiple comparisons test.

ns = non-significant, $p > 0.05$; * = $p \leq 0.05$; ** $p \leq 0.01$; *** $p \leq 0.001$; **** $p \leq 0.0001$. Scale bar, 10 μm unless otherwise specified.

Figure S4

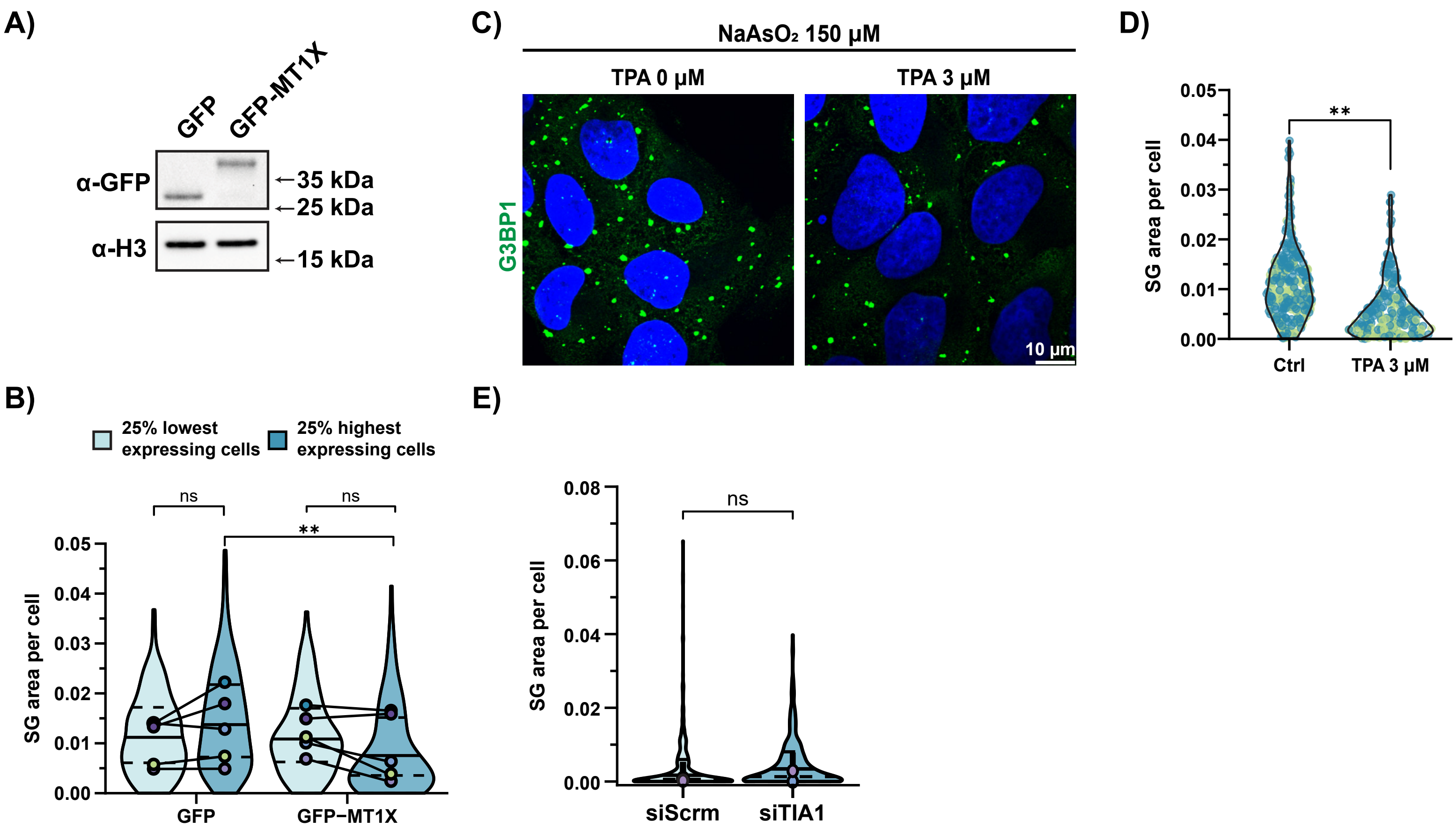
A)
GFP
GFP-MT1X
α-GFP
α-H3
35 kDa
25 kDa
15 kDa
B)
25% lowest expressing cells
25% highest expressing cells
ns
ns
**
SG area per cell
0.05
0.04
0.03
0.02
0.01
0.00
GFP
GFP−MT1X
C)
NaAsO₂ 150 μM
TPA 0 μM
TPA 3 μM
G3BP1
10 μm
D)
SG area per cell
**
Ctrl
TPA 3 μM
E)
SG area per cell
0.08
0.06
0.04
0.02
0.00
ns
siScrm
siTIA1

Figure S4: Changes to endogenous levels of free zinc affect stress granules, related to Figure 3

(A) Immunoblot of the two cell lines shows expression level of GFP and GFP-MT1X, with H3 serving as a loading control. Representative of three independent replicates.

(B) Quantification of total stress granule area normalized to the cell's cytoplasm area of the 25th and 75th percentile of GFP expressing cells. Replicate means are denoted as large dots. Averages from 5 independent experiments with a minimum of 15 cells per condition were analyzed with paired, two-tailed t-tests.

(C) Immunofluorescence of G3BP1 in U-2 OS cells treated with subjected to 150 μM arsenite stress with or without 3 μM TPA. G3BP1 in green, nuclear stain in blue.

(D) Quantification of total stress granule area per cell normalized to cell cytoplasm area. From each replicate n = 15 cells were randomly sampled and analyzed with Welch's two-tailed t-test.

(E) Quantification of total stress granule area per cell normalized to cell cytoplasm area for stressed cells treated with scrambled siRNA or TIA1 siRNA and no added zinc. Two replicates were pooled and analyzed with Welch's two-tailed t-test.

ns = non-significant, $p > 0.05$; * = $p \leq 0.05$; ** $p \leq 0.01$. Scale bar, 10 μm unless otherwise specified.

Figure S5

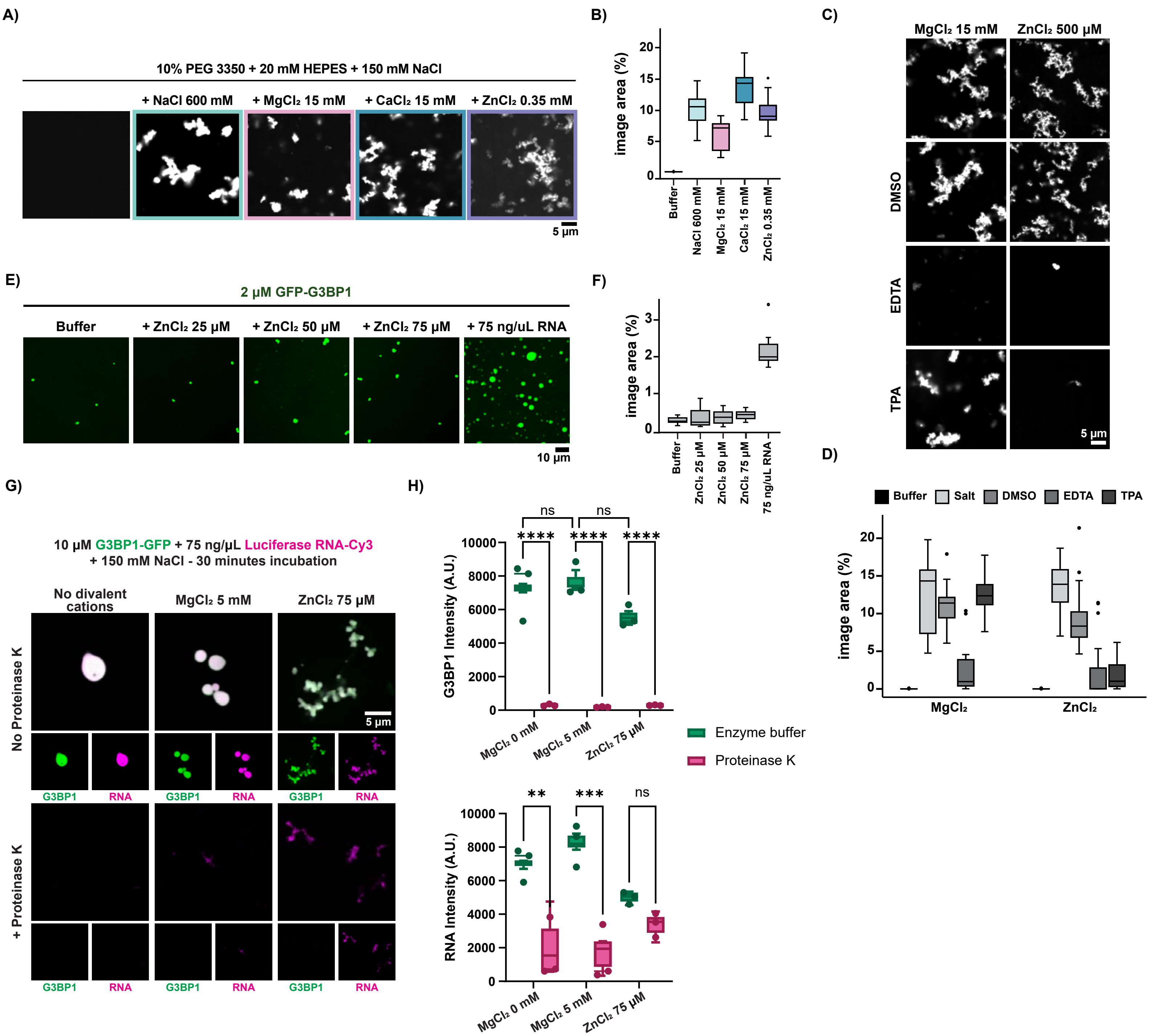
A)
10% PEG 3350 + 20 mM HEPES + 150 mM NaCl
+ NaCl 600 mM
+ MgCl₂ 15 mM
+ CaCl₂ 15 mM
+ ZnCl₂ 0.35 mM
5 µm
B)
image area (%)
Buffer
NaCl 600 mM
MgCl₂ 15 mM
CaCl₂ 15 mM
ZnCl₂ 0.35 mM
C)
MgCl₂ 15 mM
ZnCl₂ 500 µM
DMSO
EDTA
TPA
5 µm
D)
Buffer
Salt
DMSO
EDTA
TPA
image area (%)
MgCl₂
ZnCl₂
E)
2 µM GFP-G3BP1
Buffer
+ ZnCl₂ 25 µM
+ ZnCl₂ 50 µM
+ ZnCl₂ 75 µM
+ 75 ng/uL RNA
10 µm
F)
image area (%)
Buffer
ZnCl₂ 25 µM
ZnCl₂ 50 µM
ZnCl₂ 75 µM
75 ng/uL RNA
G)
10 µM G3BP1-GFP + 75 ng/µL Luciferase RNA-Cy3
+ 150 mM NaCl - 30 minutes incubation
No divalent cations
MgCl₂ 5 mM
ZnCl₂ 75 µM
No Proteinase K
+ Proteinase K
G3BP1
RNA
5 µm
H)
G3BP1 Intensity (A.U.)
RNA Intensity (A.U.)
ns
****
**
***
MgCl₂ 0 mM
MgCl₂ 5 mM
ZnCl₂ 75 µM
Enzyme buffer
Proteinase K

Figure S5: Zinc increases RNA condensation and the stability of RNA-RNA interactions *in vitro,* related to Figure 5

(A) 150 ng/μL Cy3-*luciferase* was added to condensation buffer (20 mM HEPES pH7.5, 10% PEG and 150 mM NaCl) or condensation buffer supplemented with the specified chloride salts and imaged with fluorescent confocal microscopy after 1 h incubation at 25 ^{o}C. Scale bar, 5 μm.

(B) RNA condensates in A were masked, and size was quantified as fraction of the area of the micrograph. A minimum of 15 images across 3 independent experiments performed were analyzed. All images were background-corrected to the buffer control.

(C) RNA condensates were formed with either 500 μM $ZnCl_2$ or 15 mM $MgCl_2$ for 1h and imaged. A 2x molar equivalent of EDTA or TPA was added to condensates and immediately re-imaged without mixing or additional incubation. An equal volume of 10% DMSO was used as a dilution control. Scale bar, 5 μm.

(D) RNA condensates in C were masked, and size was quantified as fraction of the area of the micrograph. A minimum of 15 images across 3 independent experiments performed were analyzed. All images were background-corrected to the buffer control.

(E) 2 μM GFP-G3BP1 (green) recombinant protein in condensation buffer (20 mM HEPES pH7.5, 150 mM NaCl) supplemented with an increasing concentration of $ZnCl_2$ or with 75 ng/μL Cy3-*luciferase* RNA. Images were obtained with fluorescent confocal microscopy after 10 min incubation at room temperature.

(F) GFP-G3BP1 condensates were masked, and size was quantified as fraction of the area of the micrograph. Quantification of a minimum of 12 images across 3 independent experiments.

(G) GFP-G3BP1-RNA co-condensates were formed using 10 μM G3BP1 and 75 ng/μL cy3-labeled *luciferase* RNA in condensation buffer (i) supplemented with 5 mM $MgCl_2$ (ii) or 75 μM $ZnCl_2$ (iii). GFP-G3BP1 in green and RNA-Cy3 in magenta.

(H) G3BP1 (top) and RNA (bottom) intensities in assemblies (arbitrary units) in enzyme buffer controls and proteinase K treated samples. Averages from 3 independent experiments with a minimum of 4 images per condition were analyzed with one-way ANOVA, corrected with Tukey's multiple comparisons test.

ns = non-significant, $p > 0.05$; * = $p \leq 0.05$; ** $p \leq 0.01$; *** $p \leq 0.001$; **** $p \leq 0.0001$. Scale bar, 10 μm unless otherwise specified.
